# The cytokinin efflux transporter ABCC4 participates in Arabidopsis root system development

**DOI:** 10.1093/plphys/kiae628

**Published:** 2024-12-24

**Authors:** Takuya Uragami, Takatoshi Kiba, Mikiko Kojima, Yumiko Takebayashi, Yuzuru Tozawa, Yuki Hayashi, Toshinori Kinoshita, Hitoshi Sakakibara

**Affiliations:** Graduate School of Bioagricultural Sciences, Nagoya University, Chikusa, Nagoya 464-8601, Japan; Graduate School of Bioagricultural Sciences, Nagoya University, Chikusa, Nagoya 464-8601, Japan; RIKEN Center for Sustainable Resource Science, Tsurumi, Yokohama 230-0045, Japan; RIKEN Center for Sustainable Resource Science, Tsurumi, Yokohama 230-0045, Japan; RIKEN Center for Sustainable Resource Science, Tsurumi, Yokohama 230-0045, Japan; Graduate School of Science and Engineering, Saitama University, Sakura, Saitama 338-8570, Japan; Graduate School of Science and Institute of Transformative Bio-Molecules (WPI-ITbM), Nagoya University, Chikusa, Nagoya 464-8602, Japan; Graduate School of Science and Institute of Transformative Bio-Molecules (WPI-ITbM), Nagoya University, Chikusa, Nagoya 464-8602, Japan; Graduate School of Bioagricultural Sciences, Nagoya University, Chikusa, Nagoya 464-8601, Japan; RIKEN Center for Sustainable Resource Science, Tsurumi, Yokohama 230-0045, Japan

## Abstract

The directional and sequential flow of cytokinin in plants is organized by a complex network of transporters. Genes involved in several aspects of cytokinin transport have been characterized; however, much of the elaborate system remains elusive. In this study, we used a transient expression system in tobacco (*Nicotiana benthamiana*) leaves to screen Arabidopsis (*Arabidopsis thaliana*) transporter genes and isolated *ATP-BINDING CASSETTE TRANSPORTER C4* (*ABCC4*). Validation through drug-induced expression in Arabidopsis and heterologous expression in budding yeast revealed that ABCC4 effluxes the active form of cytokinins. During the seedling stage, *ABCC4* was highly expressed in roots, and its expression was upregulated in response to cytokinin application. Loss-of-function mutants of *ABCC4* displayed enhanced primary root elongation, similar to mutants impaired in cytokinin biosynthesis or signaling, that was suppressed by exogenous *trans*-zeatin treatment. In contrast, overexpression of the gene led to suppression of root elongation. These results suggest that ABCC4 plays a role in the efflux of active cytokinin, thereby contributing to root growth regulation. Additionally, cytokinin-dependent enlargement of stomatal aperture was impaired in the loss-of-function and overexpression lines. Our findings contribute to unraveling the many complexities of cytokinin flow and enhance our understanding of the regulatory mechanisms underlying root system development and stomatal opening in plants.

## Teaser

An Arabidopsis ABC transporter gene encodes a cytokinin efflux transporter that participates in root system development and stomatal opening.

## Introduction

Cytokinins are a class of phytohormones involved in the regulation of various layers of plant growth and development, such as cell division, shoot development and regeneration, leaf senescence, and nutrient responses (Schaller et al. 2015; Kieber and Schaller 2018; Cortleven et al. 2019; Wybouw and De Rybel 2019; Sakakibara 2021). Naturally occurring cytokinins possess a prenyl side chain attached to the *N^6^* position of adenine, giving rise to various forms such as *N^6^*-(Δ^2^-isopentenyl)-adenine (iP), *trans*-zeatin (tZ), and *cis*-zeatin (cZ), each having distinct side chain structures (Mok and Mok 2001; Sakakibara 2006). Differences in the side chain structures are closely linked to activity strength, as demonstrated by iP and tZ showing a higher affinity to their receptors compared to cZ across various plant species including *Arabidopsis thaliana* (Romanov et al. 2006; Lomin et al. 2015) and *Zea mays* (Yonekura-Sakakibara et al. 2004; Lomin et al. 2011; Muszynski et al. 2020).

The first step of iP- and tZ-type cytokinin biosynthesis is catalyzed by adenosine phosphate-isopentenyltransferase (IPT) to produce iP-type nucleotide precursors, iP ribotides (iPRPs) (Kakimoto 2001; Takei et al. 2001). Then, the side chain of iPRPs is hydroxylated by cytochrome P450 monooxygenase (CYP735A) to synthesize tZ ribotides (tZRPs) (Takei et al. 2004; Kiba et al. 2013; Kiba et al. 2023). Finally, the CK-activating enzyme LONELY GUY (LOG) converts the nucleotide precursors to their active forms, iP and tZ (Kurakawa et al. 2007; Kuroha et al. 2009; Tokunaga et al. 2012). The cZ-type cytokinins are biosynthesized via the prenylation of tRNAs by tRNA-isopentenyltransferase (tRNA-IPT) and their degradation (Miyawaki et al. 2006). The iP riboside (iPR), tZ riboside (tZR), and cZ ribosides (cZR) are types of cytokinin precursors formed by the dephosphorylation of the corresponding ribotides (Wu et al. 2023; Sakakibara 2024).

Active and precursor forms of cytokinins are translocated throughout plant tissues and serve as cell-to-cell and organ-to-organ signaling molecules. For instance, in the development of root vascular tissue, cytokinins produced by LOG, including LOG3 and LOG4 in xylem precursor cells, are transferred to adjacent procambial cells, thereby orchestrating cell division (Ohashi-Ito et al. 2014; De Rybel et al. 2014). In shoot apical meristems, root-borne cytokinin precursors, such as tZR, that are transported to the most apical cell layer are activated by LOG4 and LOG7 (Yadav et al. 2009; Chickarmane et al. 2012; Osugi et al. 2017). Cytokinins are then perceived by receptors expressed in inner tissues, including the organizing center (Chickarmane et al. 2012; Gruel et al. 2016; Sakakibara 2021). On the other hand, root-borne tZ translocated via the xylem is mainly involved in leaf-size maintenance (Osugi et al. 2017). Whereas cytokinins and related molecules exhibit some degree of cell permeability, the dynamics of cytokinin flow in plants cannot be explained solely by simple diffusion. Although the basic framework of genes responsible for cytokinin biosynthesis and metabolism has mainly been elucidated (Osugi and Sakakibara 2015; Kojima et al. 2023; Sakakibara 2024), genes governing cytokinin flow remain relatively unexplored.

Genes involved in several aspects of cytokinin translocation have been characterized (Zhang et al. 2023). Four types of cytokinin transporters have been reported, including PURINE PERMEASE (PUP), EQUILIBRATIVE NUCLEOSIDE TRANSPORTER (ENT), AZA-GUANINE RESISTANT (AZG), and ATP-BINDING CASSETTE (ABC) TRANSPORTER. In the PUP family, PUP8 and PUP14 in *A. thaliana* and OsPUP4 in *Oryza sativa* function in the plasma membrane and play a role in transporting cytokinins (Zürcher et al. 2016; Xiao et al. 2019; Hu and Shani 2023). Tonoplast-localized PUP7 and PUP21 can act as vacuolar cytokinin importers (Hu et al. 2023). Suppression of *PUP14* expression expanded the cytokinin signaling domain in shoot apical meristems. In contrast, the simultaneous knockdown of *PUP7*, *PUP8*, and *PUP21* narrowed the signaling domain, suggesting the involvement of *PUP*s in the regulation of apoplastic cytokinin pools to modulate perception at the plasma membrane (Zürcher et al. 2016; Hu and Shani 2023). In addition, PUP1 and PUP2 have been characterized as transporters involved in cytokinin import using a heterologous yeast system, although their physiological role has not been elucidated (Gillissen et al. 2000; Bürkle et al. 2003). In the ENT family, ENT3, ENT6 and ENT8 in *A. thaliana*, and OsENT2 in *O. sativa* are thought to be localized in the plasma membrane and involved in the transport of riboside precursors (Hirose et al. 2005; Sun et al. 2005; Hirose et al. 2008). AZG1 and AZG2, members of the AZG family in *A. thaliana*, have distinct cellular localizations. AZG1 is localized solely to the plasma membrane, whereas AZG2 is found in the plasma membrane and the endoplasmic reticulum (ER) (Tessi et al. 2021; Tessi et al. 2023; Xu et al. 2024). Both proteins have been implicated in the transport of cytokinins and the regulation of root growth. In the ABC transporter family, ABCG14 in *A. thaliana* and OsABCG18 in *O. sativa* are localized to the plasma membrane and involved in long-distance transport from roots to shoots and the distribution of cytokinins and their precursors in shoots (Ko et al. 2014; Zhang et al. 2014; Zhao et al. 2019; Zhao et al. 2021a; Zhao et al. 2023). ABCG11 in *A. thaliana* has also been characterized for its involvement in modulating cytokinin responses, potentially directly or indirectly contributing to cytokinin transport (Yang et al. 2022). Furthermore, the SUGARS WILL EVENTUALLY BE EXPORTED TRANSPORTER (SWEET) HvSWEET11b in developing grains of *Hordeum vulgare* has recently been shown to transport tZ, tZR, and sugars (Radchuk et al. 2023). Although these studies are informative in understanding certain aspects of cytokinin flow within plants, it is apparent that additional transporters are essential for governing cytokinin distribution.

In this study, we searched for cytokinin transport genes using a heterologous expression system and isolated a C-type ABC transporter gene, *ABCC4,* as a candidate cytokinin efflux transporter. A loss-of-function mutation and overexpression altered the root growth profile, suggesting that ABCC4 plays a role in regulating root growth and development. Additionally, we also found that the cytokinin-dependent enlargement of stomatal aperture was impaired in the loss-of-function and overexpression lines. Our findings contribute valuable insight toward understanding the intricate flow of cytokinins in plants.

## Results

### Screening of Arabidopsis genes possibly involved in cytokinin transport

**Figure 1.**
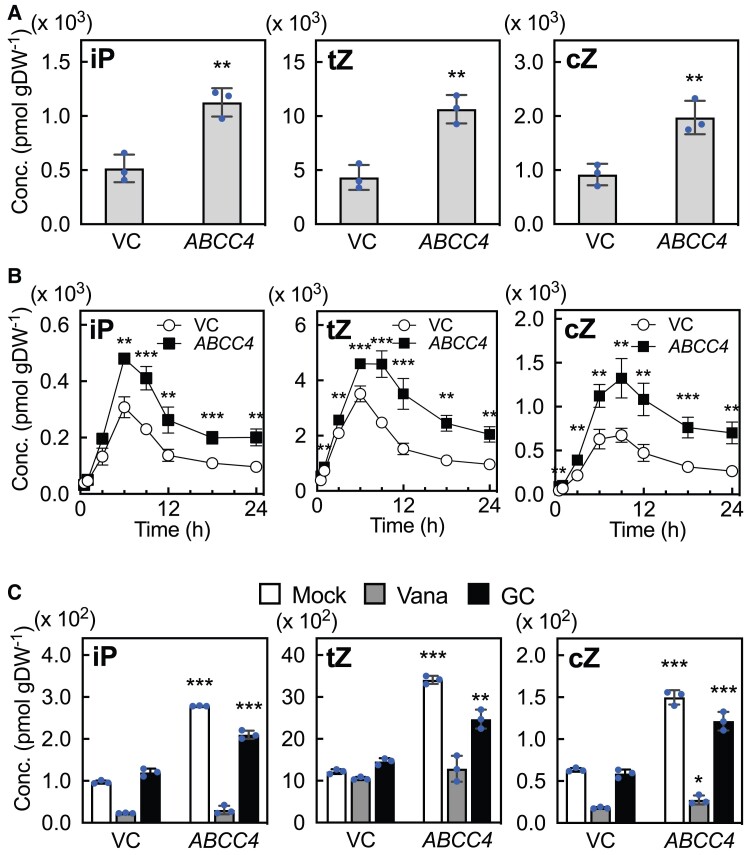
Quantification of exported cytokinins from *ABCC4*-overexpressing tobacco leaf cells. **A)** Tobacco leaf disks expressing *ABCC4* were incubated in an incubation buffer for 12 h, followed by measurements of cytokinin levels in the buffer. Data are means ± SD (*n* = 3). Asterisks represent the Student's *t*-test significance compared with VC (**P < 0.01). **B)** Time course analysis of the exported cytokinins. The leaf disks were incubated for the indicated times, and the cytokinin levels in the buffer were quantified. Data are means ± SD (*n* = 4). Asterisks represent the Student's *t*-test significance compared with VC (*P < 0.05, **P < 0.01, ***P < 0.001). **C)** The effect of ABC transporter inhibitors on the levels of exported cytokinins from *ABCC4*-overexpressing tobacco leaf cells. Tobacco leaf disks expressing *ABCC4* were incubated in incubation buffer without any inhibitors (Mock), with 1 mm orthovanadate (Vana), or with 0.1 mM glibenclamide (GC) for 12 h, after which cytokinins in the buffer were quantified. Data are means ± SD (*n* = 3). Asterisks in this figure represent the Student's *t*-test significance compared with VC (*P < 0.05, **P < 0.01, ***P < 0.001). VC, empty vector control; Conc., concentration; gDW^−1^, grams per dry weight; iP, *N^6^*-(Δ^2^-isopentenyl)-adenine; tZ, *trans*-zeatin; cZ, *cis*-zeatin.

To pursue genes potentially involved in cytokinin transport in Arabidopsis, we conducted a screening using the tobacco syringe agroinfiltration and liquid chromatography-mass spectrometry (TSAL) method (Zhao et al. 2021b). In this approach, candidate genes were transiently expressed in tobacco leaf cells under the control of the cauliflower mosaic virus (CaMV) 35S promoter, followed by quantifying the concentration of cytokinins in the cellular incubation buffer. Given the abundance of transporter genes in the Arabidopsis genome, we selected genes for screening using transcriptome data reported by Yadav et al. (2009). Specifically, we chose 61 genes that were differentially expressed in domains of the shoot apical meristem and were annotated as putative plasma membrane-localized proteins and transporters with gene ontology terms (Supplementary Table S1). This screening revealed that the expression of *ABCC4* (At2g47800), a member of the C-type ABC transporter family (Kang et al. 2011), significantly enhanced the accumulation of cytokinins, namely iP, tZ and cZ, compared to the vector control (Fig. 1A). Time course analysis showed a significantly higher accumulation of these cytokinins in the incubation buffer of *ABCC4*-expressing line compared to that of the control, and the difference between them was the highest from 6 to 12 h, and was still maintained at 24 h (Fig. 1B). Additionally, we observed increased accumulation of the riboside and ribotide precursors, albeit to a lower extent than for the corresponding cytokinins (Supplementary Fig. S1, A and B).

To validate the involvement of ABC transporter activity in the observed effect, we employed orthovanadate, a widely used phosphate analog for inhibiting phosphatases and ABC transporters. Although the presence of orthovanadate affected the level of all cytokinins and precursors in the incubation buffer of both the vector control and *ABCC4*-expressing tobacco leaves, the inhibitor clearly diminished the enhanced accumulation of cytokinins in the *ABCC4*-expressing tobacco leaves (Fig. 1C and Supplementary Fig. S2). In treatments with glibenclamide, an inhibitor of ABC transporters (Payen et al. 2001), the accumulation of all cytokinins was increased in the incubation buffer of *ABCC4*-expressing tobacco leaves; however, the extent of increase was distinctly reduced by the inhibitor treatment (Fig. 1C and Supplementary Fig. S2). Collectively, these results suggest that introduced ABCC4 plays a role in the transport of cytokinins and/or their precursors in the tobacco leaf transient expression system.

### Characterization of ABCC4 as an efflux transporter of cytokinins

**Figure 2.**
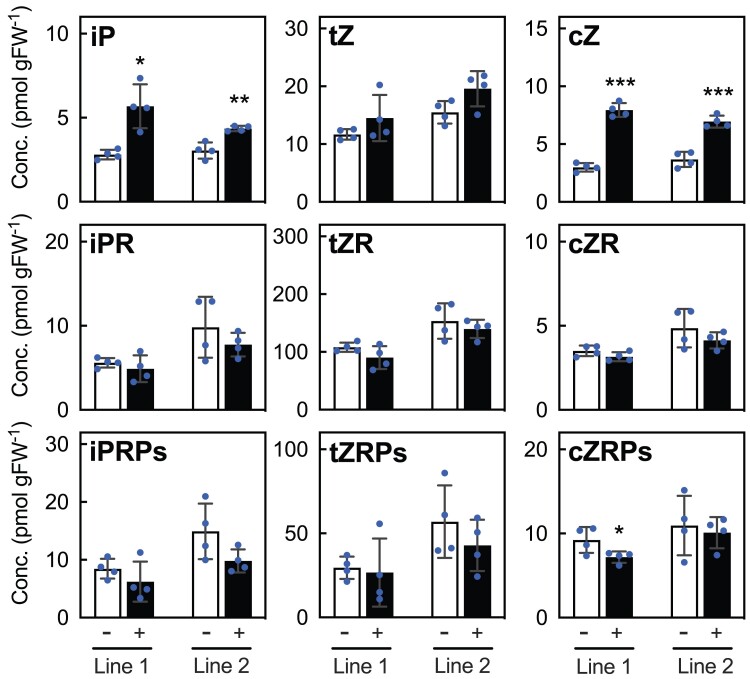
Quantification of exported cytokinins from *ABCC4*-overexpressing Arabidopsis seedlings. Seedlings of two independent transgenic Arabidopsis lines (Line 1 and Line 2) expressing *ABCC4* under control of the β-estradiol-inducible promoter were treated with (+) or without (−) 10 *μ*M of β-estradiol for 24 h, followed by measurements of cytokinins in the culture medium. Data are means ± SD (*n* = 4). Asterisks in this figure represent the Student's *t*-test significance compared with the mock (−) treatment (*P < 0.05, **P < 0.01, ***P < 0.001). Conc., concentration; gFW^−1^, grams per fresh weight; iP, *N^6^*-(Δ^2^-isopentenyl)-adenine; iPR, iP riboside; iPRPs, iP ribotides; tZ, *trans*-zeatin; tZR, tZ riboside; tZRPs, tZ ribotides; cZ, *cis*-zeatin; cZR cZ riboside; cZR, cZ ribotides.

To further verify the role of ABCC4 in the transport of cytokinins and/or their precursors, we generated transgenic Arabidopsis lines expressing *ABCC4* under the control of a β-estradiol (βE)-inducible promoter (Zuo et al. 2000). Seedlings were incubated in MS medium in the presence and absence of βE, and the levels of cytokinins and their precursors in the medium were subsequently analyzed. In comparison to the transgenic lines without βE treatment, those with βE treatment exhibited a significant increase of iP and cZ concentrations in the medium (Fig. 2). The levels of tZ showed an upward trend with βE treatment, the difference did not reach statistical significance. On the other hand, levels of the corresponding ribosides and ribotides either remained unchanged or decreased with βE treatment (Fig. 2).

**Figure 3.**
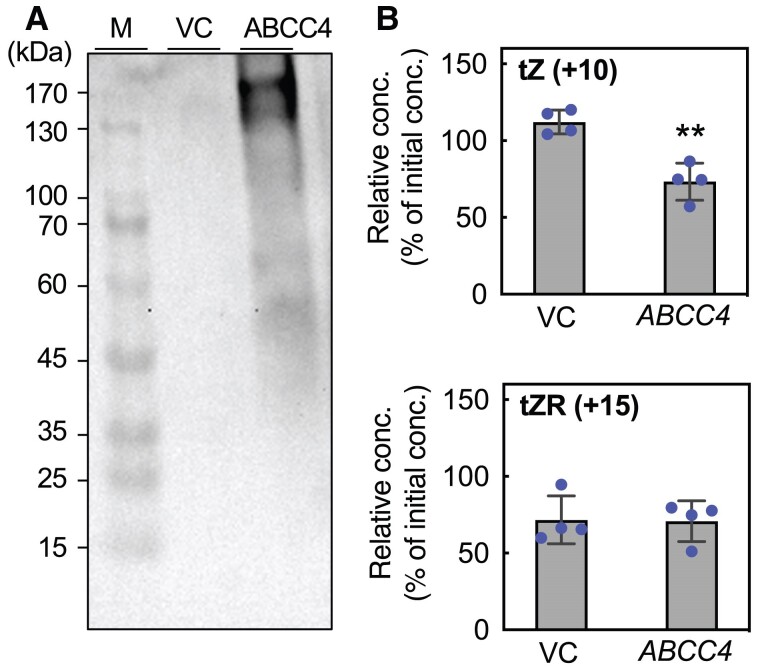
Heterologous expression of ABCC4 in yeast cells. **A)** Immunoblot detection of ABCC4 in yeast cells. Yeast (strain YPH499) harboring the pYES-empty vector (VC) or pYES-ABCC4 (ABCC4) was cultured, and total protein was extracted. Total proteins (60 *μ*g) were subjected to immunoblot analysis using an anti-ABCC4 antibody. Sizes of the molecular mass markers (M) are indicated on the left. **B)** Cytokinin transport assay in yeast. VC and ABCC4 yeast strains treated with stable isotope-labeled 50 nm tZ (+10) or tZR (+15) were incubated in isotope free-buffer for 0 and 10 min, followed by quantification of the labeled compounds in the cells. The relative concentration was calculated by defining the concentration at 0 min as 100%. Data are means ± SD (*n* = 4). Asterisks in this figure represent Student's *t*-test significance compared with VC (**P < 0.01). conc., concentration; tZ, *trans*-zeatin; tZR, tZ riboside.

In experiments using whole plants, it is difficult to determine genuine transport substrates because cytokinins can be derivatized by their metabolic enzymes. Therefore, we conducted a heterologous transport assay employing a budding yeast, *Saccharomyces cerevisiae*. In a yeast strain expressing *ABCC4* under the control of a galactose-inducible promoter, a protein band corresponding to the estimated size of ABCC4 (169 kDa) was detected (Fig. 3A). We then quantified the intracellular cytokinin levels in the yeast strain that had been fed previously with stable isotope-labeled tZ (+10) or tZR (+15), whose molecular masses exceed those of authentic tZ and tZR by 10 and 15 Da, respectively. As a result, the levels of tZ (+10) in yeast cells expressing *ABCC4* were significantly lower in comparison to those observed in the empty vector control (Fig. 3B). In contrast, no significant difference was found in the tZR (+15) levels. These results strongly support the hypothesis that ABCC4 is involved in the efflux transport of cytokinins.

### Expression analysis of *ABCC4*

ABCC4 was initially isolated as MULTIDRUG RESISTANCE-ASSOCIATED PROTEIN 4 (AtMRP4) and characterized as a transporter involved in the regulation of stomatal aperture (Klein et al. 2004). In that study, AtMPR4 was localized to the plasma membrane, and the expression was found by RT-PCR and GUS staining to be widespread throughout the plant, including the basal region of hypocotyls, primary roots, guard cells, and sepals (Klein et al. 2004). To gain more insight into the expression patterns of *ABCC4*, we conducted reverse transcription (RT)-quantitative PCR (RT-qPCR) analyses.

**Figure 4.**
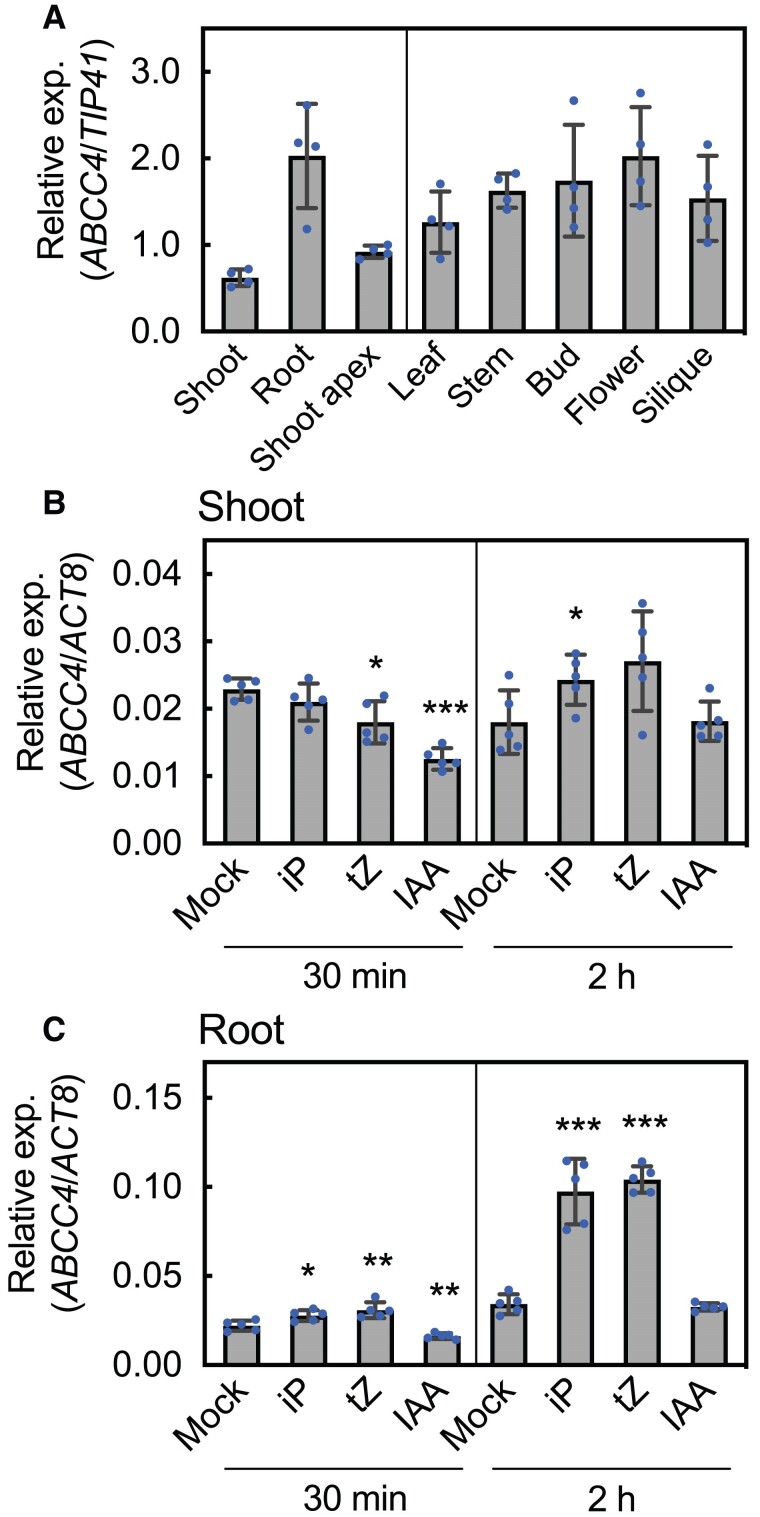
Expression patterns of *ABCC4* in Arabidopsis. **A)** Expression patterns of *ABCC4* in plant organs. Total RNAs were extracted from shoots and roots of 10-d-old Arabidopsis seedlings (left panel) and from the indicated organs of 45-d-old plants (right panel) and subjected to RT-qPCR analysis. Expression levels of *ABCC4* were normalized to that of *TIP41,* a housekeeping gene. Data are means ± SD (*n* = 4). **B, C)** Effect of cytokinin and auxin treatments on the expression of *ABCC4* in shoots **B)** and roots **C)** Arabidopsis seedlings grown for 10 d on 1/2 agar plates were sprayed with 0.01% dimethyl sulfoxide (Mock),1 *μ*M *N^6^*-(Δ^2^-isopentenyl)-adenine (iP), 1 *μ*M *trans*-zeatin (tZ), or 0.4 *μ*M indole-3-acetic acid (IAA). The shoots and roots were separately harvested after 30 min and 2 h. Total RNAs were extracted and subjected to RT-qPCR analysis. Expression levels of *ABCC4* were normalized to that of *ACT8.* Data are means ± SD (*n* = 5). Asterisks in this figure represent Student's *t*-test significance compared with Mock (*P < 0.05, **P < 0.01, ***P < 0.001). exp., expression.

*ABCC4* exhibited higher expression in the roots than the shoots and shoot apices during the seedling phase and showed broad expression across all above-ground organs during the adult and reproductive phases (Fig. 4A). Since the expression of transporter genes is often regulated by its transport substrates (Jasiński et al. 2001; Swarup et al. 2008; Ko et al. 2014; Pierman et al. 2017; Zhao et al. 2019), we examined the expression of *ABCC4* in response to cytokinin treatment. Following exposure of Arabidopsis seedlings to tZ, no remarkable changes in the *ABCC4* expression were found in shoots at either 30 min or 2 h post-treatment (Fig. 4B). This same lack of responsiveness of *ABCC4* expression to tZ was also observed in the guard cell-enriched samples (Supplementary Fig. S3). On the other hand, *ABCC4* expression in roots was significantly upregulated at 30 min after cytokinin treatment, with expression levels reaching approximately three times higher than that of the mock treatment at 2 h post-treatment (Fig. 4C). These results align with the hypothesis that *ABCC4* is involved in transporting cytokinins. Indole-3-acetic acid (IAA) treatment resulted in the transient suppression of *ABCC4* expression within 30 min (Fig. 4, B and C).

### Characterization of an *abcc4* loss-of-function mutant

To explore the physiological role of *ABCC4*, we obtained a T-DNA insertional loss-of-function mutant line (*abcc4-1*) (Supplementary Fig. S4A) and generated a genome-edited frame-shift mutant line (*abcc4-2*) by the CRISPR/Cas9 mediated genome editing technique (Supplementary Fig. S5). RT-PCR analysis confirmed the absence of full-length *ABCC4* transcripts in the *abcc4-1* mutant (Supplementary Fig. S4B).

**Figure 5.**
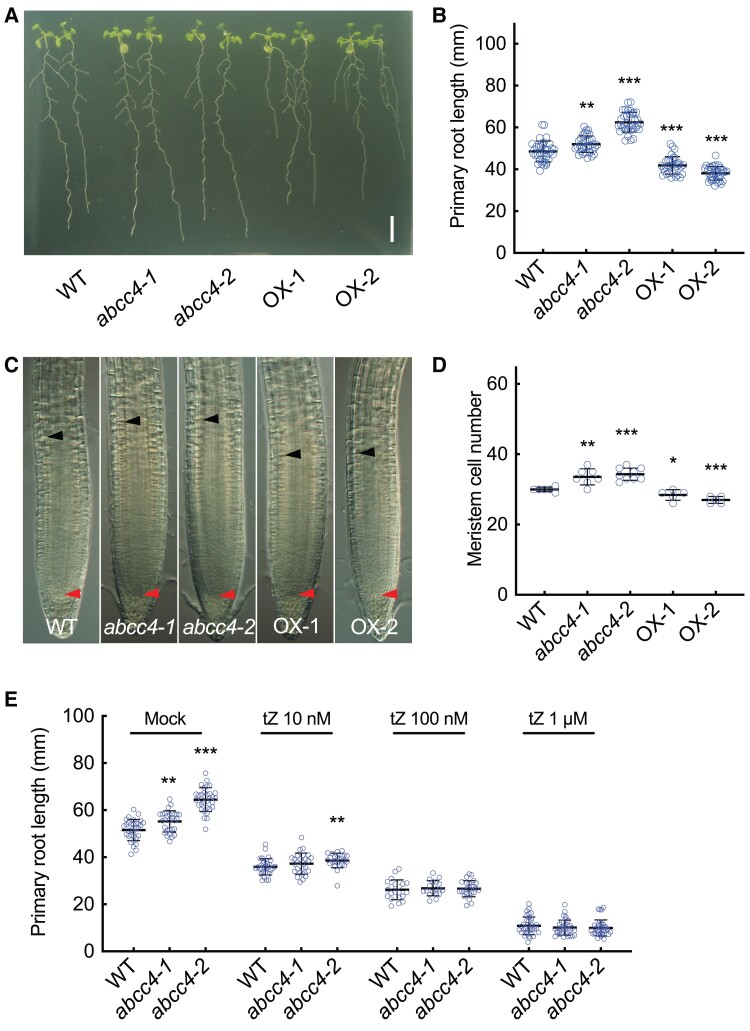
Root growth phenotypes of *abcc4* mutants and *ABCC4*-overexpression lines. **A)** A representative image of WT (Col-0), a T-DNA insertion mutant (*abcc4-1*), a genome-edited mutant (*abcc4-2*), and two *ABCC4*-overexpression lines (OX-1 and OX-2). The plants were grown on 1/2MS agar plates for 12 d. Scale bar, 1 cm. **B)** Primary root length of WT, *abcc4-1*, *abcc4-2*, OX-1, and OX-2 seedlings grown on 1/2MS agar plates for 14 d. Data are means ± SD (*n_WT_* = 37, *n_abcc4-1_* = 34, *n_abcc4-2_* = 36, *n_OX-1_* = 33, *n_OX-2_* = 36). **C)** Representative images of the root meristem of WT, *abcc4-1*, *abcc4-2*, OX-1 and OX-2 seedlings grown for 6 d. Red and black arrowheads indicate the quiescent center and the boundary of first elongated cortex cell, respectively. **D)** Root meristem cell number of WT, *abcc4-1*, *abcc4-2*, OX-1 and OX-2 seedlings grown for 6 d. Data are means ± SD (*n_WT_* = 6, *n_abcc4-1_* = 7, *n_abcc4-2_* = 9, *n_OX-1_* = 5, *n_OX-2_* = 5). **E)** The effect of cytokinin treatment on primary root length of WT, *abcc4-1*, and *abcc4-2.* Seedlings were grown on 1/2MS agar medium with 0.01% DMSO (Mock) or the indicated concentration of *trans*-zeatin (tZ) for 11 d. Data are means ± SD (*n_mock-WT_* = 32, *n_mock-abcc4-1_* = 29, *n_mock-abcc4-2_* = 32, *n_tZ10nM-WT_* = 32, *n_tZ10nM-abcc4-1_* = 26, *n_tZ10nM-abcc4-2_* = 25, *n_tZ100nM-WT_* = 21, *n_tZ100nM-abcc4-1_* = 18, *n_tZ100nM-abcc4-2_* = 25, *n_tZ1 µM-WT_* = 36, *n_tZ1 µM-abcc4-1_* = 34, *n_tZ1 µM-abcc4-2_* = 35). Asterisks in this figure represent Student's *t*-test significance compared with WT (**P < 0.01, ***P < 0.001).

The primary roots of the loss-of-function mutants grown on 1/2MS agar plates were significantly more elongated compared to the wild type (WT) (Fig. 5, A and B). Since cytokinins are known as crucial signaling molecules controlling root growth rate by determining root meristem cell number (Beemster and Baskin 2000; Werner et al. 2003; Dello Ioio et al. 2007; Moubayidin et al. 2010), we measured the number of meristematic cells in primary roots and found that their number had increased in *abcc4* (Fig. 5, C and D). On the other hand, no apparent differences were observed in shoot growth (Supplementary Fig. S6) or lateral root growth (Fig. 5A and Supplementary Fig. S7) when compared to the WT. To assess the impact of the mutation on cytokinin levels, we analyzed cytokinin concentrations in whole roots of 10-d-old *abcc4* seedlings; however, no consistent changes in the mutants compared with WT were observed (Supplementary Fig. S8A).

Next, we investigated the effects of cytokinin treatment on primary root growth in the mutants. The length of WT primary roots declined with increasing concentrations of tZ. Primary root length of the mutants also decreased with tZ treatment, but the elongated root phenotype of the mutants was nearly abolished at 10 nM and was eliminated at higher concentrations of 100 nM and 1 *µ*M (Fig. 5E). These results suggest that cytokinin is relevant to the primary root phenotype of the mutant.

### Analysis of phylogenetically related homologs of *ABCC4*

To investigate genes potentially sharing functions in cytokinin transport with ABCC4, we selected *ABCC14*, the phylogenetically closest homolog (82% identity) (Kang et al. 2011), and evaluated its cytokinin transport activity. However, no cytokinin transport capacity was detected by the TSAL method or the βE-induced expression system in Arabidopsis. We generated an *abcc4 abcc14* double mutant by the CRISPR/Cas9 system (Supplementary Fig. S5), but root and shoot growth were not different in the double mutant compared with the *abcc4* single mutant (Supplementary Fig. S9).

### Characterization of *ABCC4*-overexpression lines

To gain further insights into the physiological role of *ABCC4*, we generated Arabidopsis plants overexpressing *ABCC4* driven by the CaMV 35S promoter and measured their expression levels (Supplementary Fig. S10A). No discernible morphological changes were found in the shoots of overexpressors grown in soil (Supplementary Fig. S6). However, the root meristem cell number and the primary root growth of two independent *ABCC4* overexpressing lines were reduced (Fig. 5, A to D). Additionally, these overexpressors exhibited higher lateral root density than the WT without altering the total density of lateral root primordia (LRP) and lateral roots (Supplementary Fig. S7, A and B). Furthermore, the elongation rate of lateral roots was increased (Supplementary Fig. S7C), suggesting that the emergence of lateral roots was promoted in the overexpressors.

Primary root elongation and lateral root development are reportedly regulated by auxin and cytokinin (Blilou et al. 2005; Dello Ioio et al. 2007; Laplaze et al. 2007; Marhavý et al. 2014; Du and Scheres 2018). Therefore, we hypothesized that the levels and/or actions of auxin and/or cytokinin might be altered in the overexpressors. Consequently, we quantified IAA, cytokinins, and their precursor levels and examined the expression levels of auxin- and cytokinin-response marker genes by RT-qPCR in whole roots. Although the level of iPRPs was slightly lower, the level of IAA and all other cytokinins and their precursors in roots of *ABCC4* overexpressors was not altered (Supplementary Fig. S8). Furthermore, no significant differences were detected in the expression levels of *INDOLE-3-ACETIC ACID INDUCIBLE 5* and *19* (*IAA5* and *IAA19*, respectively) and type-A *ARABIDOPSIS RESPONSE REGULATOR 5, 6,* and *15* (*ARR5, ARR6,* and *ARR15*, respectively) in overexpressors compared to the WT (Supplementary Fig. S10, B and C).

**Figure 6.**
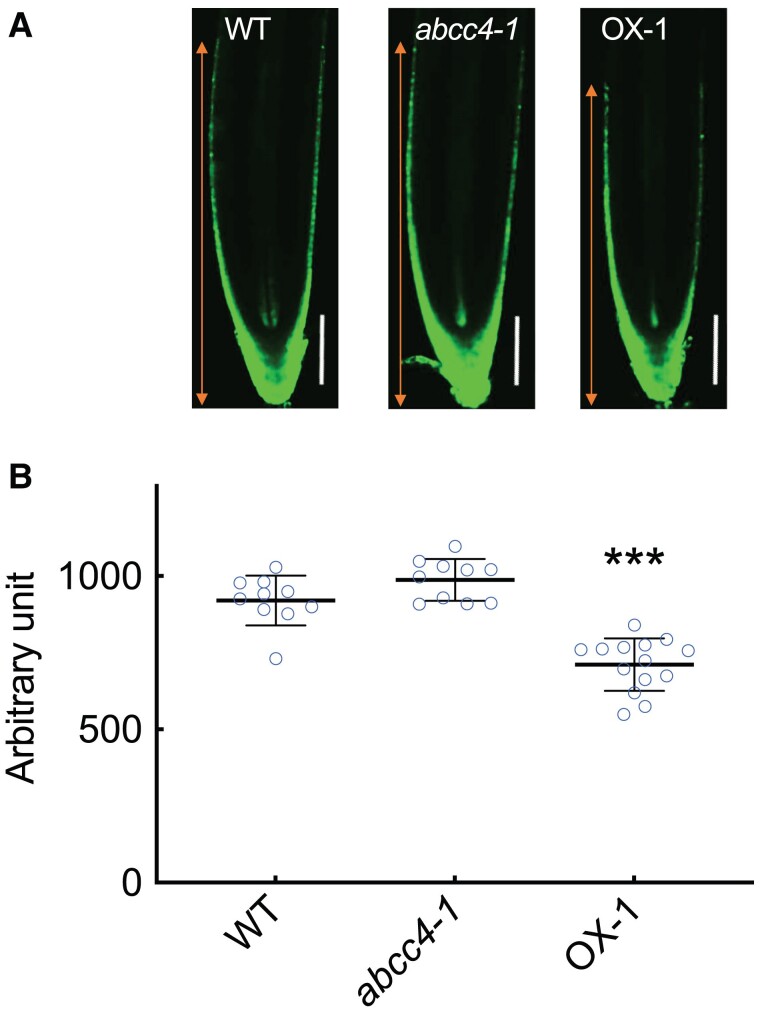
Comparison of *TCSn:GFP* fluorescent pattern in the root of WT, *abcc4* mutant and *ABCC4-*overexpression lines. **A)** Representative fluorescent images of WT (Col-0), *abcc4-1*, and *ABCC4*-overexpression line (OX-1) grown for 10 d. Scale bar, 100 *µ*m. Arrowhead line in orange shows the measured GFP fluorescence. **B)** The range of GFP fluorescence observed in the epidermal tissue from the root tip was measured by ImageJ. Data are means ± SD (n*_WT_* = 10, n*_abcc4-1_* = 10, n*_OX-1_* = 14). Asterisks represent Student's *t*-test significance compared with WT (***P < 0.001).

To investigate the effects of *ABCC4* loss-of-function and overexpression on cytokinin signaling at the tissue and cellular levels, we generated transgenic *abcc4* and *ABCC4*-overexpression lines harboring *TCSn:GFP* (Zürcher et al. 2013) and conducted microscopic observations of the fluorescence patterns in the primary root. The results showed that the range of GFP fluorescence in the epidermal tissue of the root tips was reduced in the overexpression lines compared to the WT. While the range in the *abcc4-1* was larger than that of WT, the difference was not statistically significant (Fig. 6). These results suggest that *ABCC4* modulates cytokinin action in the root tip region.

### Examination of *ABCC4* involvement in root-to-shoot cytokinin transport

Given the involvement of some cytokinin transporters in organ-to-organ cytokinin transport (Ko et al. 2014; Zhang et al. 2014; Zhao et al. 2023), we conducted experiments to compare the ability of the WT, *abcc4*, and *ABCC4* overexpressors to transfer tZ from roots to shoots. The expression levels of *ARR5* in the shoots were analyzed after exogenous tZ application to the roots. As a control, we used *abcg14,* a mutant known to be impaired in root-to-shoot cytokinin transport (Ko et al. 2014; Zhang et al. 2014). The expression level of *ARR5* remained unchanged in *abcg14* upon exposure to tZ, as previously reported (Ko et al. 2014), but increased in the WT, *abcc4-1*, and *ABCC4* overexpressor (Supplementary Fig. S11). These results suggest that *ABCC4* is not essentially involved in root-to-shoot cytokinin transport.

### Effect of the *abcc4* mutation on stomatal aperture

**Figure 7.**
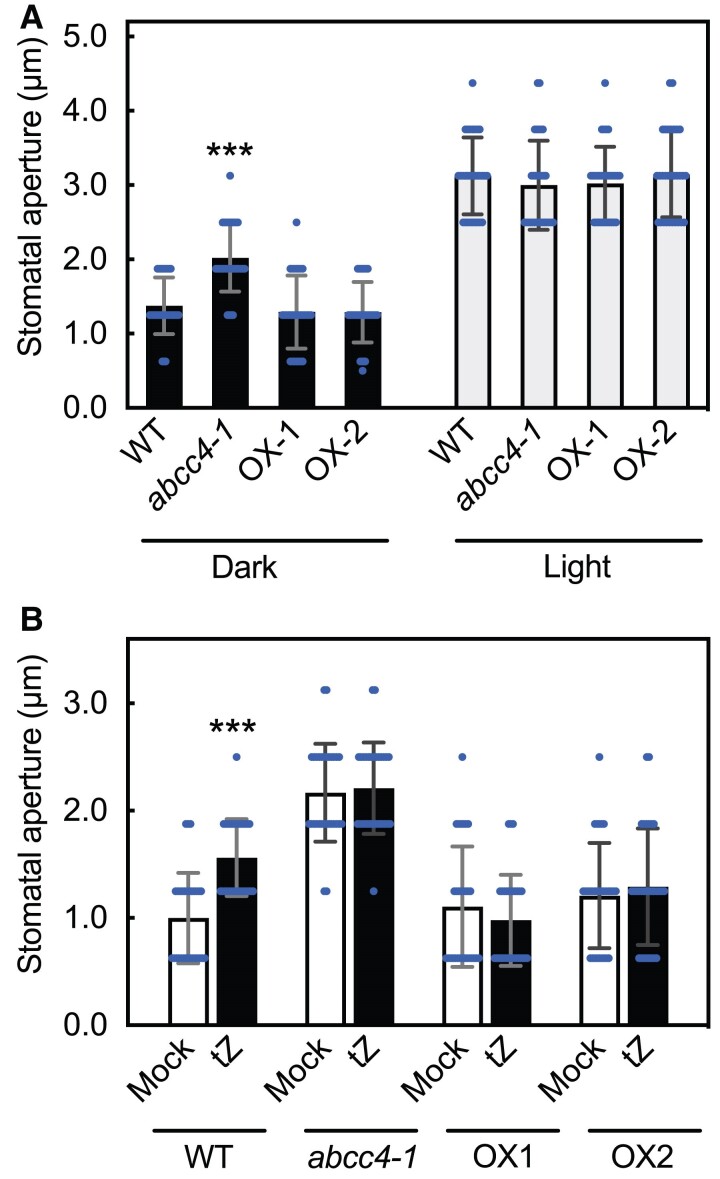
Stomatal aperture in WT, *abcc4* mutant and *ABCC4-*overexpression lines. **A)** Measurement of stomatal aperture in WT (Col-0), *abcc4-1*, OX-1 and OX-2 in the dark or light. Detached leaves were placed in dark (Dark) or lighted (Light) conditions for 2 h. Data are means ± SD (*n* = 30). Asterisks represent Student's *t*-test significance compared with WT (***P < 0.001). **B)** Effect of *trans*-zeatin (tZ) application on the stomatal aperture in the dark. Detached leaves of WT, *abcc4-1*, OX-1 and OX-2 were placed in dark for 3 h followed by 0.01% DMSO (Mock) or 1 *μ*M tZ (tZ) treatment. Data are means ± SD (*n* = 30). Asterisks represent Student's *t*-test significance compared with Mock (***P < 0.001).

A previous study showed that disruption of *ABCC4* (*AtMPR4*) in the Ws-2 and L*er* backgrounds resulted in larger stomatal apertures in light and dark conditions (Klein et al. 2004). In our analysis using Columbia as the genetic background, the *abcc4* mutant also showed increased stomatal apertures in the dark compared to the WT, whereas no difference was found in lighted conditions (Fig. 7A). In the overexpressors, no distinction was noted between dark and lighted conditions.

To further elucidate the role of *ABCC4* in cytokinin action and stomatal aperture regulation, we treated rosette leaves with tZ and measured the stomatal aperture. The results indicated that tZ treatment significantly increased stomatal aperture in the WT, while it did not have a significant effect in the *abcc4-1* mutant, with the size of stomatal aperture remaining high under both mock and tZ treatments. No significant change was observed in the overexpression lines compared to the WT (Fig. 7B). These findings suggest that *ABCC4* plays a role in controlling stomatal aperture in response to cytokinin treatment.

## Discussion

In this study, we have identified and characterized *ABCC4* as a gene encoding a cytokinin efflux transporter in Arabidopsis. The cytokinin transport function was evaluated using the TSAL system, a conditional expression system in Arabidopsis, and a yeast transport assay. We also detected cytokinin efflux in the empty vector control over time in the TSAL system (Fig. 1B and Supplementary Fig. S1B). This phenomenon has been previously reported (Zhao et al. 2021b) and suggests that some experimental Agrobacterium strains possess the ability to produce cytokinins even in the absence of the biosynthesis gene on the Ti plasmid.

Identifying a cytokinin efflux transporter is noteworthy, as efflux transporters of cytokinins have been comparatively less characterized than influx transporters (Zürcher et al. 2016; Tessi et al. 2023, 2021; Hu et al. 2023). However, we were unable to examine differences in transport properties among cytokinin species, such as iP, tZ, and cZ, since competitive experiments for *in planta* exporter analysis are challenging to execute. We have attempted in vitro translation and reconstitution using liposomes (Nozawa et al. 2020) but have not yet obtained conclusive results. Other experimental systems will be required for a more in-depth analysis, including *Xenopuslaevis* oocyte assays.

To determine the expression sites of *ABCC4* at the tissue and cellular levels, we generated two types of transgenic Arabidopsis lines harboring *ABCC4pro:GUS* constructs, including one based on a previous study (Klein et al. 2004). However, GUS staining was undetectable in both cases. As an alternative approach, we referred to publicly available transcriptome data at the tissue (Brady et al. 2007; Yang et al. 2008) and single-cell levels (Ryu et al. 2019) by using the ePlant database (https://bar.utoronto.ca/eplant/) (Winter et al. 2007; Waese et al. 2017). The root tissue-level data indicated that *ABCC4* is primarily expressed in the elongation and maturation zones of the root, rather than in the meristematic zone (Supplementary Fig. S12A). Single-cell transcriptome analysis further showed that *ABCC4* is mainly expressed in early differentiating epidermal cells and cortical cells (Supplementary Fig. S12B).

We found that loss-of-function mutants of *ABCC4* had elongated primary roots, whereas overexpression of *ABCC4* resulted in shortened primary roots (Fig. 5, A to D). Although no significant differences in cytokinin concentration were detected at the whole-root level (Supplementary Fig. S8A), the overexpression line demonstrated altered cytokinin action in tissue-level analysis using *TCSn:GFP* (Fig. 6). This suggests that *ABCC4* plays a role in proper distribution of cytokinin in the root tip region.

Increased cytokinin action typically leads to inhibition of primary root growth (To et al. 2004; Zhang et al. 2011). The higher expression of *ABCC4* in differentiating cells and in the elongation zone, as compared to the apical meristem, suggests a minor role of *ABCC4* in meristematic tissues. Instead, ABCC4-mediated cytokinin efflux seems to be important in the regulation of intracellular and extracellular cytokinin levels in the transition from meristem to elongation phases, which influences root system development. A previous study showed that cytokinin signaling in the transition zone controls root meristem size through the degradation of cell cycle regulators (Takahashi et al. 2013). This could provide a model to explain the observed phenotypes in our study. Specifically, *abcc4* loss-of-function mutant may experience reduced cytokinin concentrations in the apoplast of the transition zone, suppressing cytokinin signaling. This suppression might hinder the transition to the endocycle, causing an enlarged root apical meristem (Dello Ioio et al. 2008; Takahashi et al. 2013). Supporting this model, the longer primary root phenotype in *abcc4* mutants was rescued by external tZ application (Fig. 5E). However, it is also possible that the change in the *TCSn:GFP* fluorescence range is a secondary consequence of the altered meristem size.

Although cytokinins are considered to be perceived at both the ER membrane and plasma membrane (Caesar et al. 2011; Lomin et al. 2011; Wulfetange et al. 2011; Romanov et al. 2018; Antoniadi et al. 2020; Kubiasová et al. 2020), a study has also suggested that cytokinin receptors are preferentially localized to the plasma membrane to perceive apoplast cytokinins in root meristematic cells (Kubiasová et al. 2020). To further elucidate the role of *ABCC4* in root development, a more detailed understanding of the subcellular localization of the receptors and its function in the transition zone is necessary.

In addition to the primary root phenotype, the lateral root density and the lateral root elongation rate of the overexpressors were increased (Supplementary Fig. S7). Given that no alterations in lateral root growth and development were observed in the *abcc4* mutant, we assume that the lateral root phenotype of the overexpressors is irrelevant to the inherent function of *ABCC4*. Instead, the phenotype could potentially be an indirect consequence of the reduced primary root growth since it has long been known that removal or damage to the primary root results in increased lateral root formation in many plants (Thimann 1936; Xu et al. 2017).

A previous study provided compelling evidence for the involvement of *ABCC4* (*AtMPR4*) in regulating stomatal opening (Klein et al. 2004). The loss-of-function mutants in Wassilewskija and Landsberg ecotype backgrounds had larger stomatal apertures than the WT (Klein et al. 2004). Our analysis of *abcc4* in the Columbia background also showed enlarged stomatal apertures compared to the WT (Fig. 7A), indicating that *ABCC4* plays a role in controlling stomatal aperture across different genetic backgrounds. Cytokinins have been implicated in promoting stomatal opening (Das et al. 1976; Tanaka et al. 2006), and *ABCC4* is expressed in guard cells (Klein et al. 2004)(Supplementary Fig. S12C). Our analysis showed that the loss-of-function and overexpression of *ABCC4* abolished cytokinin-responsive stomatal aperture change, indicating a potential relevance of the ABCC4's cytokinin exporter activity to the stomatal phenotype. A possible explanation of the result is that cytokinin concentration in the guard cells of the *abcc4-1* mutant was elevated, causing stomata to remain maximally open, thus rendering them unresponsive to external cytokinin. In contrast, in the overexpression lines, high cytokinin efflux activity likely counteracted the applied cytokinin, resulting in a lack of response. This result also suggests that cytokinin perception in guard cells is primarily intracellular, with receptors on the ER membrane mediating cytokinin action. Nonetheless, our current understanding of cytokinin action on stomata remains limited, necessitating further investigation in future studies.

Overall, this study revealed the multifaceted roles of *ABCC4* in plant growth and development. Additional studies are needed to elucidate the precise mechanisms underlying the effects of *ABCC4* on cytokinin flow in roots, as well as its impact on root growth and stomatal aperture. Nevertheless, our findings provide a foundation for future research to unravel the complex interplay between cytokinin distribution, perception, and growth and development.

## Materials and methods

### Plant materials and growth conditions

*Nicotiana benthamiana* plants were grown in a commercial soil mixture (Supermix A, Sakata) at 23 °C under long-day conditions (16-h light/8-h dark) with a photosynthetic photon flux density of 125 *µ*mol m^−2^ s^−1^. *A. thaliana* ecotype Columbia (Col-0) was used as the WT. The *abcg14* mutant has been characterized previously (Ko et al. 2014). The T-DNA insertion line SALK_090215 (*abcc4-1*) was obtained from the Arabidopsis Biological Resource Center (https://abrc.osu.edu/), and its genotype was determined by genomic PCR using primers shown in Supplementary Table S2 (Supplementary Fig. S4). *A. thaliana* seeds were sterilized and germinated on Murashige-Skoog (MS) agar plates (0.8% agar and 1% sucrose), 1/2 MS agar plates (1.1% agar and 1% sucrose), or on soil (Supermix A) at 22 °C under long-day conditions (16-h light/8-h dark) with a photosynthetic photon flux density of 45 to 80 *µ*mol m^−2^ s^−1^.

### Plasmid construction

The coding region of *ABCC4* and *ABCC14* (with a stop codon) was amplified by RT-PCR with specific primers (Supplementary Table S2) and cloned into the pENTR/D-TOPO vector (Invitrogen) to generate an entry vector, pENTR-ABCC4. After confirmation of the sequence, the entry vector and the Gateway LR Clonase II enzyme mix (Invitrogen) were used to integrate the ABCC4 coding region into Gateway binary vectors pER8-GW-HA (Zuo et al. 2000) or pBA002-GW, a derivative of pBA002-GFP (Kiba et al. 2018), to generate pER8-ABCC4 or pBA002-ABCC4, respectively. To generate the pYES-ABCC4 plasmid for yeast transport assays, *ABCC4* was cloned into the Gateway binary vector pYES-DEST52 using pENTR-ABCC4 and Gateway LR Clonase II enzyme mix (Invitrogen).

### Agroinfiltration-based transporter activity assay in tobacco

Transient expression of genes-of-interest in tobacco leaf cells was performed according to the method described by Zhao et al. (2021b). Leaves of 30-d-old tobacco plants were infiltrated with *Agrobacterium tumefaciens* strain C58C1 harboring pBA002-ABCC4. Four days after infiltration, the leaves were cut into 3 mm × 3 mm square samples, washed twice with efflux buffer (5 mM MES-KOH buffer, pH 5.7), and then incubated in 6 mL incubation buffer (5 mM MES-KOH buffer, pH 5.7) at 22 °C for 12 h. Aliquots of the buffer were used for cytokinin quantification. The incubated leaves were dried and weighed. For the treatment with inhibitors, either glibenclamide (final conc. 0.1 mm) or orthovanadate (final conc. 1 mm) was added to the incubation buffer.

### Cytokinin and auxin quantification

Cytokinins and auxin were semi-purified with solid-phase extraction columns and quantified using an ultra-performance liquid chromatography (UPLC)-tandem quadrupole mass spectrometer (ACQUITY UPLC System/XEVO-TQXS; Waters Corp.) with an octadecylsilyl column (ACQUITY UPLC HSS T3, 1.8 µm, 2.1 mm × 100 mm, Waters Corp.) as described (Kojima et al. 2009).

### Generation of transgenic lines

Transgenic plants were generated by the *Agrobacterium tumefaciens*-mediated floral dip method (Clough and Bent 1998) using EHA105 strain harboring pER8-ABCC4, pER8-ABCC14, pBA002-ABCC4 or pMg137_ABCC4ABCC14. Transformants were selected on MS agar plates containing 5 *μ*g L^–1^ bialaphos sodium salt (for pBA002) or 25 *μ*g L^–1^ hygromycin (for pER8).

### Transporter activity assay in an Arabidopsis conditional expression system

The transporter activity assay was performed according to a previously described method (Ohyama et al. 2008). Arabidopsis seeds of β-estradiol-inducible *ABCC4* and *ABCC14* overexpression lines (T2 generation) were sown directly in 30 mL of MS liquid medium and cultured with rotation (140 rpm) under continuous light (130 *µ*mol m^−2^ s^−1^) at 22 °C. After 6 d, the culture medium was exchanged with new MS. After another 1-d culture, transgene expression was induced with 10 *μ*M estradiol for 24 h. Aliquots of the culture medium were subjected to cytokinin quantification.

### Synthesis of stable isotope-labeled cytokinins

tZ(+10) was synthesized from commercially available (^13^C_10_, ^15^N_5_)-adenosine 5′-monophosphate (AMP(+15)) and hydroxymethylbutenyl pyrophosphate (HMBDP) by a series of enzymatic reactions. First, AMP(+15) and HMBDP were catalyzed by recombinant Tzs (Krall et al. 2002) to form tZRMP(+15). Calf intestine alkaline phosphatase (TaKaRa) was added to the reaction mixture to generate tZR(+15). tZR(+15) was de-ribosylated by purine-nucleoside phosphorylase DeoD from *Escherichia coli* to form tZ(+10) (Takei et al. 2003). The tZ(+10) and tZR(+15) were purified using HPLC (model Alliance 2695; Waters) linked to a photodiode array detector (2996; Waters) on a reverse-phase column (SymmetryC18, 5 *µ*m, 4.6 × 150 mm Cartridge; Waters).

### Transporter activity assay in yeast cells

pYES and pYES-ABCC4 were introduced into the YPH499 yeast strain using a Fast Yeast Transformation Kit (Geno Technology). Transformed yeast cells were grown under selective conditions in a minimal medium (46.7 g L^−1^ Minimal Sd Agar Base (TaKaRa), 0.78 g L^−1^ uracil dropout supplement (TaKaRa)). Cytokinin transport assays were performed according to the method described by Zhao et al. (2023). The yeast cells were pre-cultured in a liquid yeast medium (2% raffinose) and re-suspended in an induction medium (1% raffinose and 2% galactose). After 18 h of induction, the yeast cells were incubated with an uptake buffer (100 mM potassium phosphate buffer, pH 5.8) supplemented with 50 nm tZ (+10) or tZR (+15) at 28 °C for 20 min. After washing with uptake buffer, the yeast cells were suspended and incubated in an uptake buffer for 0 and 10 min at 28 °C. Cells were collected by centrifugation and subjected to cytokinin extraction and quantification. The cytokinin concentration at 0 min (initial concentration) was defined as 100%.

### Immunoblot analysis

Yeast protein was extracted by homogenizing yeast cells with acid-washed glass beads (Sigma-Aldrich) in homogenizing medium (0.25 m sorbitol, 50 mM Tris-acetate pH7.5, 2 mM EGTA-Tris, 1% PVP-40, 2 mM DTT, 0.5×protease inhibitor cocktail (Sigma-Aldrich)). The protein concentration was determined using a Bio-Rad Bradford protein assay kit (Bio-Rad), and 60 *μ*g of total protein was separated by sodium dodecyl sulfate-polyacrylamide gel electrophoresis (SDS–PAGE). The anti-ABCC4 antibodies were obtained by immunizing a rabbit with a peptide of 19 amino acid residues (Cosmo Bio) representing positions 1246 to 1263 (CKQFTDIPSESEWERKETL) of ABCC4. The yeast harboring pYES was used as the empty vector control.

### RT-qPCR analysis

Total RNA was extracted from plant samples using NucleoSpin RNA (Macherey-Nagel). Total RNA was used for RT by the ReverTra Ace qPCR RT Master Mix (Toyobo). Quantitative PCR (qPCR) was performed on a Quant Studio 3 Real-Time PCR system (Thermo Fisher) using the KAPA SYBR Fast qPCR kit (KAPA Biosystems) and gene-specific primer sets (Supplementary Table S3). Expression levels were normalized using *ACT8* or *TIP41* as internal controls (Kiba et al. 2018).

### CRISPR–Cas9 mutagenesis of *ABCC4* and *ABCC14*

The frame-shift mutants of *ABCC4* and/or *ABCC14* (Supplementary Fig. S5B) were generated using the transfer RNA-based-multiplex CRISPR–Cas9 vector, pMgPec12-137-2A-GFP (Hashimoto et al. 2018). Guide sequences for *ABCC4* were designed by CHOPCHOP (Labun et al. 2021), and multiplex CRISPR–Cas9 vectors pMg137_ABCC4ABCC14 harboring guide sequences for *ABCC4* and *ABCC14* were constructed as described (Hashimoto et al. 2018). pMg137_ABCC4ABCC14 contained guide sequences g4 and g14 (Supplementary Fig. S5A). Transgenic plants were generated by the *Agrobacterium tumefaciens*-mediated method using the EHA105 strain harboring the vectors. Mutations were identified by DNA sequencing of PCR products amplified with specific primer sets (Supplementary Table S2) and genomic DNA prepared from the transformants.

### Evaluation of growth phenotypes

Rosette diameters, primary root lengths, lateral root numbers, and range of GFP fluorescence were determined from pictures using ImageJ (https://imagej.net/ij/). The stage I to stage VII primordia were counted as LRP according to method of Malamy and Benfey (1997). LR density was calculated as the number of LR per total root length.

### Root meristem cell number analysis

The root meristem cell number for each plant was analyzed by counting the number of cortex cells in a file extending from the quiescent center to the first elongated cortex cell. Six-d-old seedlings (longer than 1 cm) viewed with a microscope (BX51; Olympus) were used to measure meristem cell numbers.

### Analysis of *abcc4* and *ABCC4*-overexpression lines harboring *TCSn:GFP*

The *abcc4-1* and *ABCC4*-overexpression line (OX-1) were crossed with Arabidopsis harboring *TCSn:GFP* (Zürcher et al. 2013). The homozygotes were observed for GFP fluorescence using a confocal laser scanning fluorescence microscope (FV1000; Olympus).

### Stomatal aperture

Stomatal aperture measurements were started in the morning after exposure to 14-16 h of darkness using plants grown for 3 wk on soil. The stomatal aperture in epidermal tissues was measured as described (Hayashi et al. 2024). Leaves collected from dark-treated plants were floated on a buffer comprising 5 mM 2-ethanesulfonic acid (MES)-BTP (pH 6.5), 20 mM KCl and 0.1 mM CaCl_2_. After a light (200 *µ*mol m^−2^ s^−1^) or dark treatment for 2 h, isolated epidermal fragments were prepared by blending the leaves for 3 s twice in Milli-Q water with a blender (Waring Commercial) at high speed. The epidermal fragments were collected on pieces of 58-μm nylon mesh and were immediately microphotographed using a microscope (BX50; Olympus). Stomatal apertures on the abaxial side of fragments were measured.

### Isolation of guard cells

Epidermal fragments including stomatal guard cells were isolated from rosette leaves of 4- to 6-wk-old Arabidopsis plants as described previously (Ando et al. 2013). Blended peels were sonicated to remove contaminating mesophyll and epidermal cells (Virlouvet and Fromm 2015). The epidermal fragments were collected on a 58-µm nylon mesh and frozen in a tube with liquid nitrogen.

### Statistical analysis

All statistical analyses were conducted using Microsoft Excel. Details of the analyses are provided in the Figure legends.

### Accession numbers

Sequence data from this article can be found in The Arabidopsis Information Resource database (http://www.arabidopsis.org) under the following accession numbers: *ABCC4* (At2g47800), *ABCC14* (At3g62700), *ACT8* (At1g49240), *ARR5* (At3g48100), *ARR6* (At5g62920), *ARR15* (At1g74890), *IAA5* (At1g15580), *IAA19* (At3g15540), and *TIP41* (At4g34270).

## Supplementary Material

kiae628_Supplementary_Data

## Data Availability

The data underlying this article are available in the article and in its online supplementary material.

## Supplementary data

The following materials are available in the online version of this article.

**Supplementary Figure S1.** Quantification of exported cytokinin precursors from *ABCC4*-overexpressing tobacco leaf cells.

**Supplementary Figure S2.** The effect of ABC transporter inhibitors on the levels of exported cytokinin precursors from ABCC4-overexpressing tobacco leaf cells.

**Supplementary Figure S3.** Expression of *ABCC4* in guard cells.

**Supplementary Figure S4.** T-DNA insertional *abcc4-1* mutant.

**Supplementary Figure S5.** The *abcc4-2* and *abcc4 abcc14* mutants generated using the CRISPR/Cas9 system.

**Supplementary Figure S6.** Shoot growth phenotypes of *abcc4* mutants and *ABCC4-*overexpression lines.

**Supplementary Figure S7.** Lateral root phenotypes of *abcc4* mutants and *ABCC4*-overexpression lines.

**Supplementary Figure S8.** Quantification of cytokinins, cytokinin precursors, and auxin in the whole roots of the *abcc4* mutants and *ABCC4* overexpressors.

**Supplementary Figure S9.** Growth phenotype of *abcc4 abcc14.*

**Supplementary Figure S10.** Expression levels of cytokinin and auxin-responsive marker genes in *ABCC4* overexpressing lines.

**Supplementary Figure S11.**  *ARR5* expression levels in shoots followed by tZ application in roots in an *abcc4* mutant and an *ABCC4* overexpressing line.

**Supplementary Figure S12.** Expression level of *ABCC4* according to the ePlant database (https://bar.utoronto.ca/eplant/).

**Supplementary Table S1.** List of 61 candidate genes.

**Supplementary Table S2.** List of primers used for vector construction and genotyping.

**Supplementary Table S3.** List of primers used for RT-qPCR analysis.

## Dive Curated Terms

The following phenotypic, genotypic, and functional terms are of significance to the work described in this paper:

PUP7 Gramene: AT4G18197
PUP7 Araport: AT4G18197

PUP8 Gramene: AT4G18195
PUP8 Araport: AT4G18195

PUP1 Gramene: AT1G28230
PUP1 Araport: AT1G28230

cis-zeatin CHEBI: CHEBI:46570

primary root AmiGo: PO:0020127

AZG1 Gramene: AT3G10960
AZG1 Araport: AT3G10960

AZG2 Gramene: AT5G50300
AZG2 Araport: AT5G50300

trans-zeatin CHEBI: CHEBI:16522

ENT3 Gramene: AT4G05120
ENT3 Araport: AT4G05120
