# Supplementary material for: The cytokinin efflux transporter ABCC4 participates in Arabidopsis root system development
Source: Plant Physiol. 2024 Dec 24;197(1):kiae628. doi: 10.1093/plphys/kiae628 (PMC11668331; doi:10.1093/plphys/kiae628)
Supplement: kiae628_Supplementary_Data [file kiae628_supplementary_data.zip › PP2024RA00759R1_Supplemental_Figures_1_12_Supplemental_Tables_1_3.pdf]

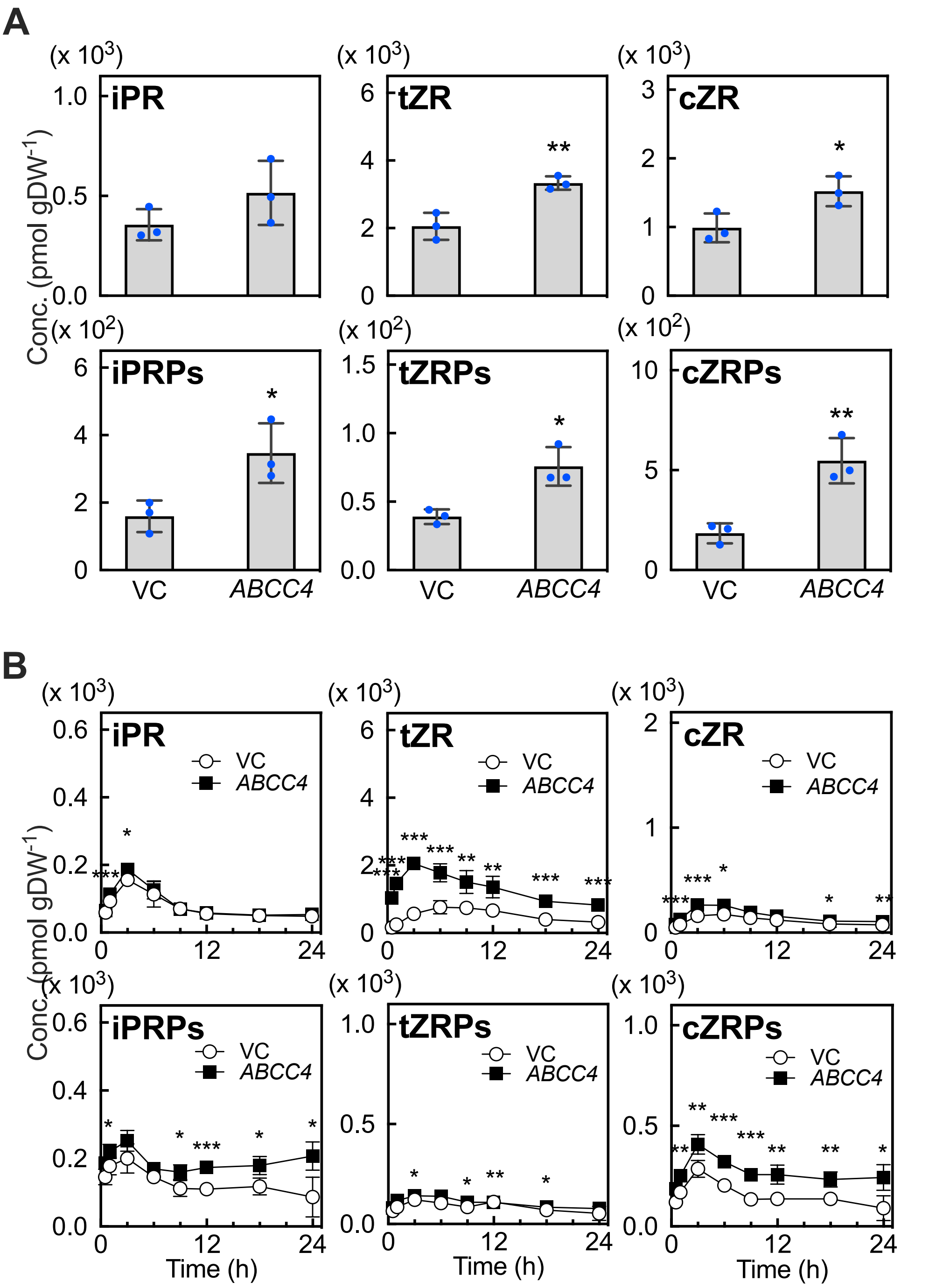

Supplementary Figure S1. Quantification of exported cytokinin precursors from *ABCC4*-overexpressing tobacco leaf cells

(A) Tobacco leaf disks expressing *ABCC4* were incubated in incubation buffer for 12 h, followed by measurement of cytokinin levels in the buffer. VC, empty vector control. Data are means ± SD (n = 3). **(B)** Time-course analysis of the exported cytokinins. Leaf disks were incubated for the indicated times, and the cytokinin levels in the buffer were quantified. Data are means ± SD (n = 4). Asterisks in this figure represent the Student's t-test significance compared with VC (*$P < 0.05$, **$P < 0.01$, ***$P < 0.001$). Conc., concentration; gDW^{-1}, grams per dry weight; iPR, N^6-(Δ^2-isopentenyl)-adenine riboside; iPRPs, iP ribotides; tZR, *trans*-zeatin riboside; tZRPs, tZ ribotides; cZR, *cis*-zeatin riboside; cZRPs, cZ ribotides.

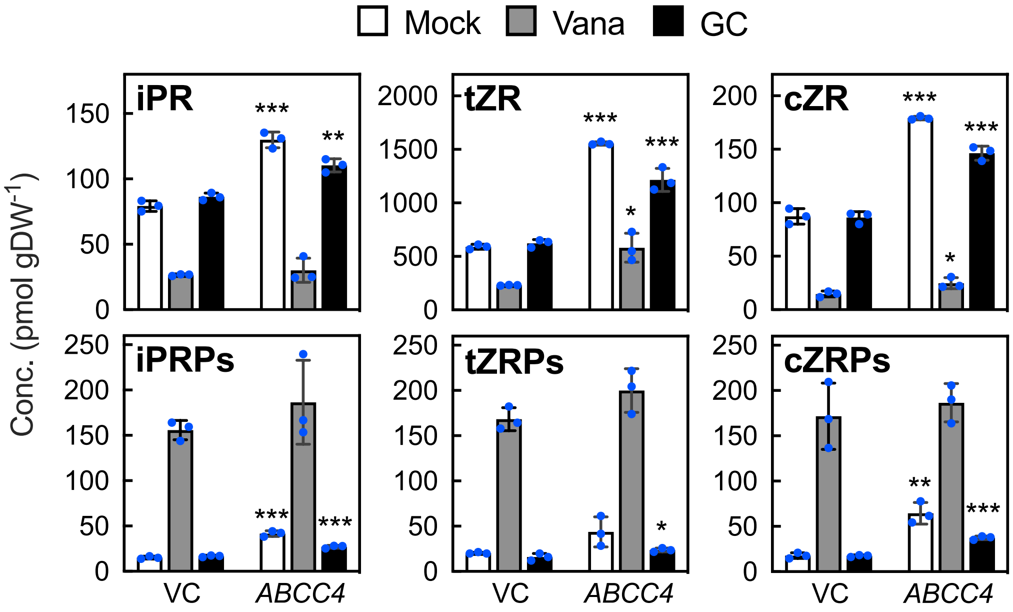

Supplementary Figure S2. The effect of ABC transporter inhibitors on the levels of exported cytokinin precursors from ABCC4-overexpressing tobacco leaf cells
Tobacco leaf disks expressing *ABCC4* were incubated in incubation buffer in the presence of 1 mM orthovanadate (Vana), 0.1 mM glibenclamide (GC), or absence (Mock) of for 12 h, followed by measurement of cytokinin precursors in the buffer. Data are means ± SD (n = 3). Asterisks represent Student's t-test significance compared with empty vector control (VC) (*P < 0.05, **P < 0.01, ***P < 0.001). Conc., concentration; gDW^{-1}, grams per dry weight. iPR, N^6-(Δ^2-isopentenyl)-adenine riboside; iPRPs, iP ribotides; tZR, *trans*-zeatin riboside; tZRPs, tZ ribotides; cZR, *cis*-zeatin riboside; cZRPs, cZ ribotides.

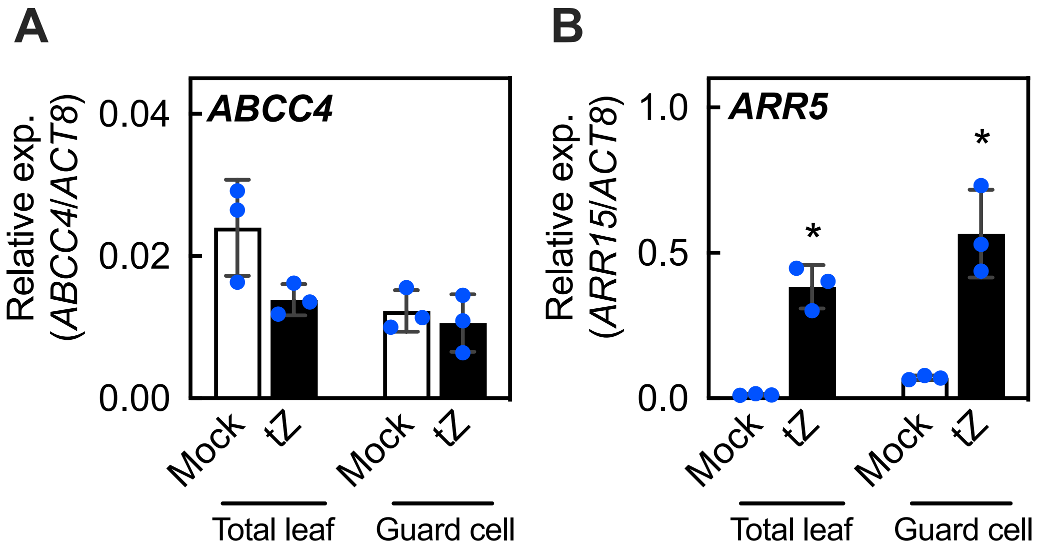

Supplementary Figure S3. Expression of *ABCC4* in guard cells
Expression levels of *ABCC4* **(A)** and *ARR5* **(B)** in total leaf and guard cells. Rosette leaves and epidermal fragments enriched in stomatal guard cells were collected 2 hours after 0.01% DMSO (Mock) or 1 µM *trans*-zeatin (tZ) spray. Total RNAs were extracted and subjected to RT-qPCR analysis. Expression levels of *ABCC4* and *ARR5* were normalized to that of *ACT8*. Data are means ± SD (n = 3). Asterisks represent Student's t-test significance compared with Mock (*$P < 0.05$). exp., expression.

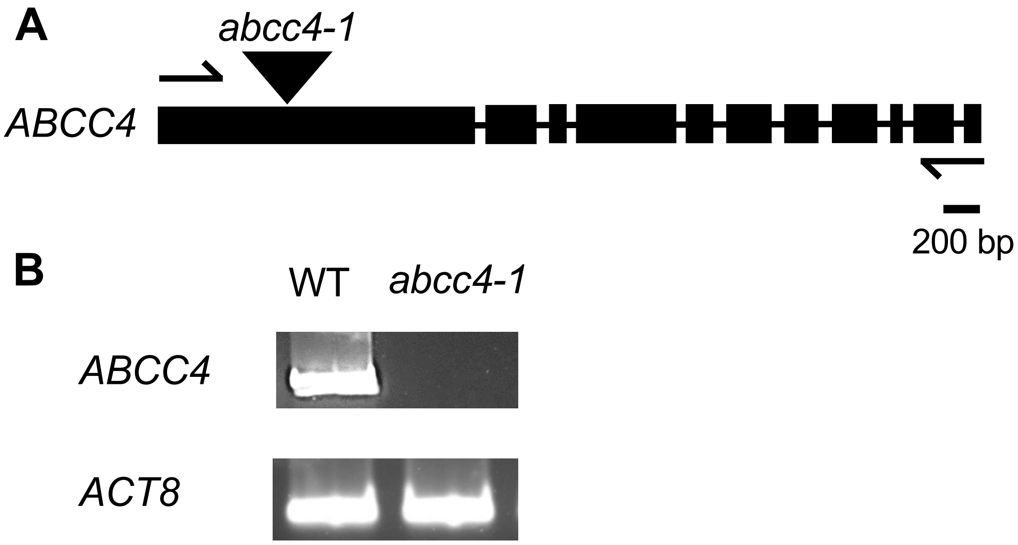

Supplementary Figure S4. T-DNA insertional *abcc4-1* mutant
(A) A diagram of the T-DNA insertional site for *abcc4-1*. Boxes represent exons, and lines represent introns. The black arrowhead shows the position of the T-DNA insertion, and the arrows denote the direction and position of the PCR primers used in (B). The scale bar indicates 200 bp. **(B)** RT-PCR detection of the *ABCC4* transcripts in WT (Col-0) and the *abcc4-1* mutant. *ACT8* was used as the reference.

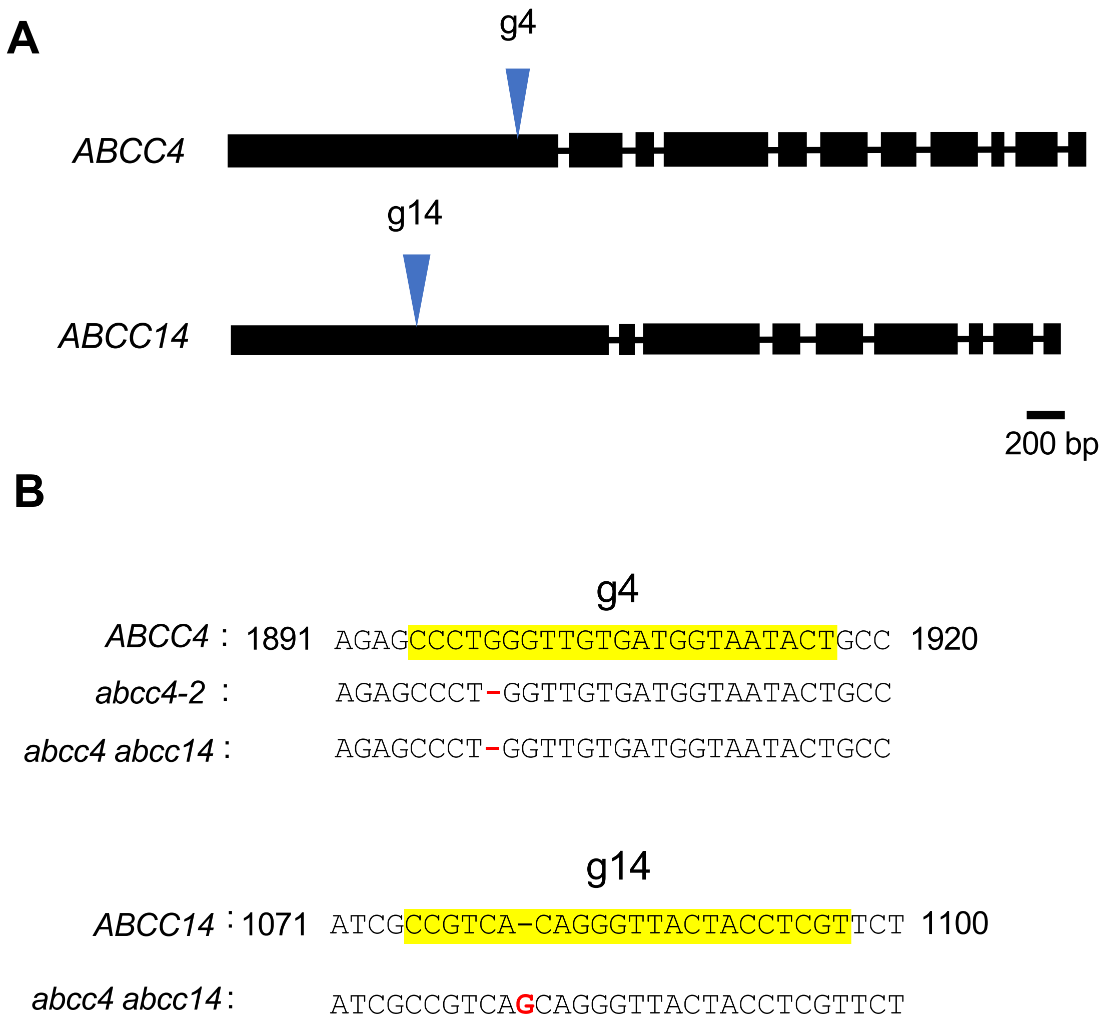

Supplementary Figure S5. The *abcc4-2* and *abcc4 abcc14* mutants generated using the CRISPR/Cas9 system
(A) Schematic representation of CRISPR target sites. Rectangular boxes represent exons, black bars represent introns, and blue triangles identify CRISPR target sites. The bar indicates a 200 bp scale. **(B)** Partial sequences of the wild-type *ABCC4*, the wild-type *ABCC14*, the *abcc4-2* mutant, and the *abcc4 abcc14* double mutant. The sequence corresponding to a guide RNA is highlighted in yellow. Numbers represent positions in a genome sequence when the first nucleotide of the putative start codon is counted as 1. The red letter and red dashes indicate an inserted and deleted sequences, respectively. The nucleotide deletion in *ABCC4* resulted in an in-frame stop codon at the 674th amino acid. The nucleotide insertion in *ABCC14* resulted in an in-frame stop codon at the 423rd amino acid.

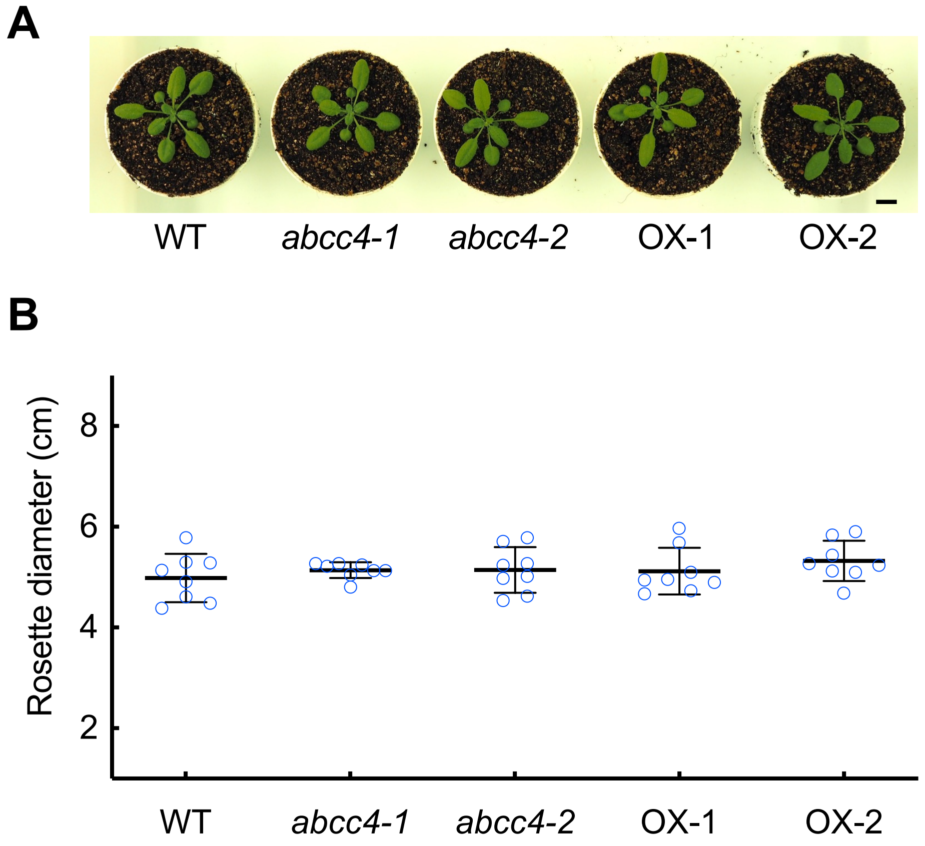

Supplementary Figure S6. Shoot growth phenotypes of *abcc4* mutants and *ABCC4* overexpression lines

(A) Representative images of WT (Col-0), *abcc4-1*, *abcc4-2*, and two *ABCC4* overexpression lines (OX-1 and OX-2) grown for 23 d on soil. Scale bar, 1 cm. **(B)** Rosette diameter of WT, *abcc4-1*, *abcc4-2*, OX-1, and OX-2 plants grown 23 d on soil. Data are means ± SD (*n = 9*).

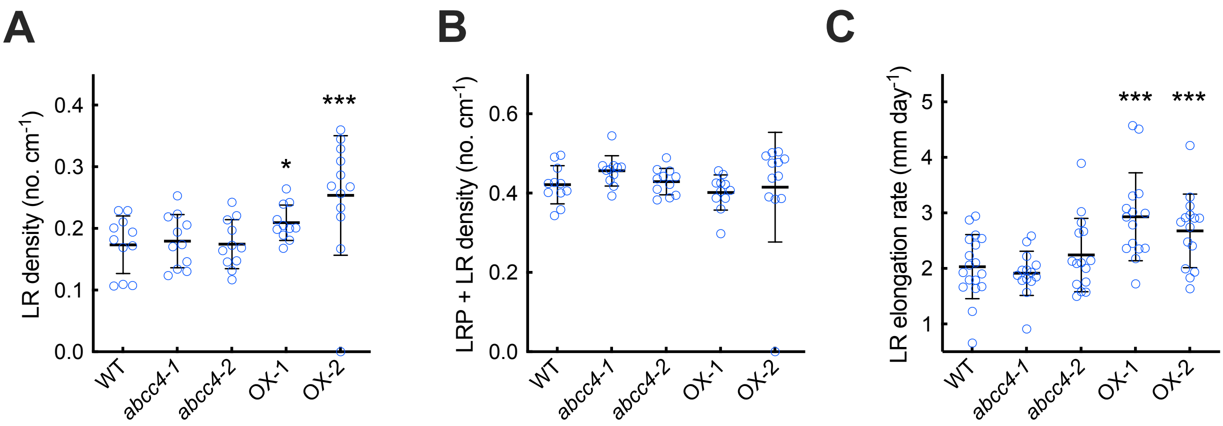

Supplementary Figure S7. Lateral root phenotypes of *abcc4* mutants and *ABCC4* overexpression lines

(A) Densities of lateral roots (LR) of WT (Col-0), *abcc4-1*, *abcc4-2*, OX-1, and OX-2 seedlings grown for 10 d. The density was calculated by dividing the number of LR with primary root length. Data are means ± SD (n=11). **(B)** Total densities of LR primordia (LRP) and LR of WT, *abcc4-1*, *abcc4-2*, OX-1, and OX-2 seedlings grown for 10 d. The sum of LRP and the LR number was divided by primary root length to calculate the total density. Data are means ± SD (n=11). **(C)** Lateral root elongation rate of WT, *abcc4-1*, *abcc4-2*, OX-1, and OX-2 seedlings. The elongation rate was calculated by subtracting the lateral root length of 10-d-old seedlings from that of 12-d-old seedlings and dividing it by the number of days elapsed. Data are means ± SD (n_{WT}= 18, $n_{abcc4\text{-}1}$= 14, $n_{abcc4\text{-}2}$= 15, $n_{OX\text{-}1}$=15, $n_{OX\text{-}2}$=15). Asterisks in this figure represent Student's *t*-test significance compared with WT (*$P < 0.05$, ***$P < 0.001$).

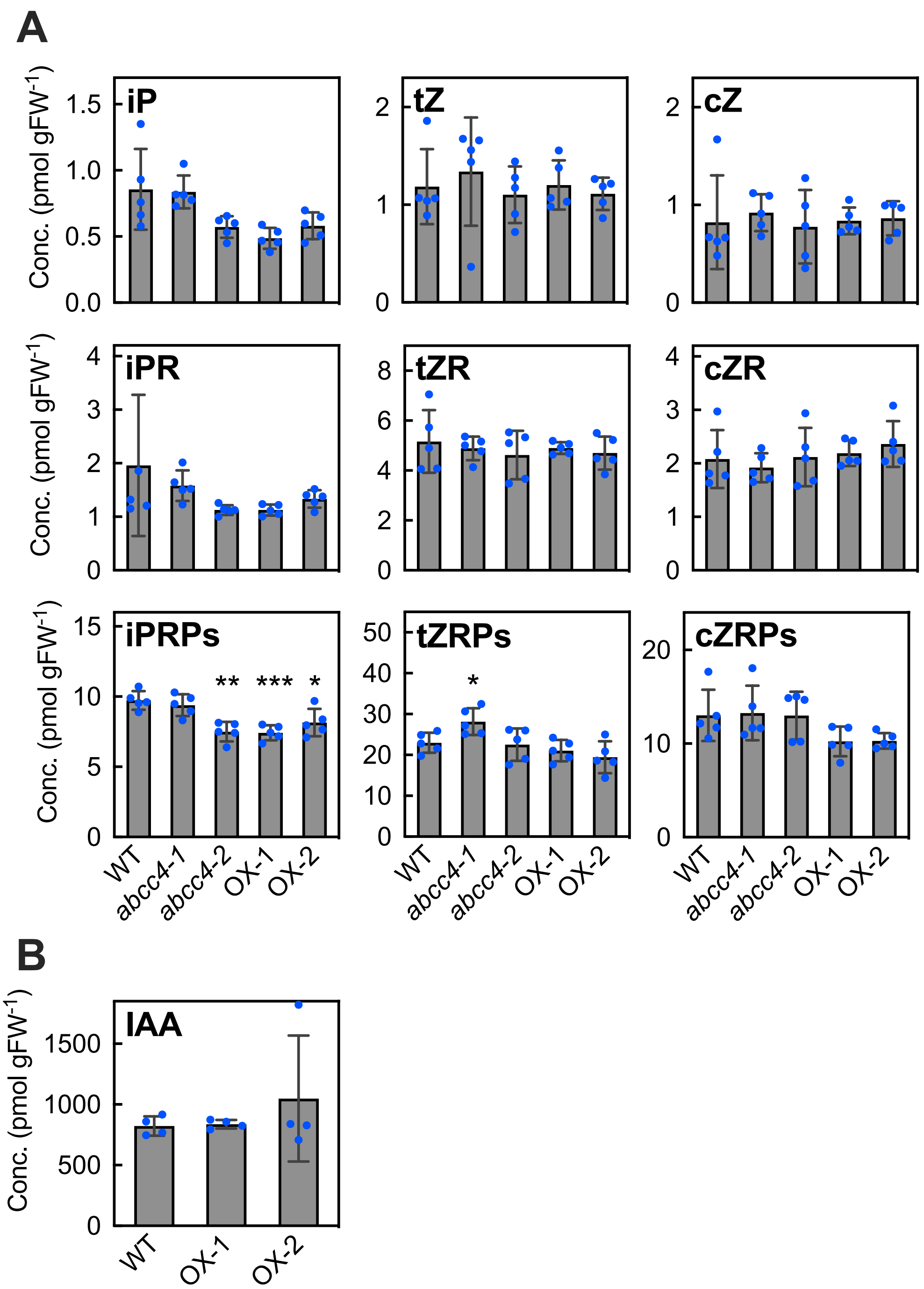

Supplementary Figure S8. Quantification of cytokinins, cytokinin precursors, and auxin in the whole roots of the *abcc4* mutants and *ABCC4* overexpressors
(A) Quantification of cytokinins and their precursors in the whole roots of WT (Col-0), *abcc4-1*, *abcc4-2*, OX-1, and OX-2 seedlings grown for 10 d on 1/2MS agar plates. Data are means ± SD (*n* = 4~5). **(B)** Quantification of indole 3-acetic acid (IAA) in the whole roots of WT, OX-1, and OX-2 seedlings grown for 7 d on 1/2MS agar plates. Data are means ± SD (*n* = 4~5). Asterisks in this figure represent Student's *t*-test significance compared with WT (**P* < 0.05, ***P* < 0.01, ****P* < 0.001). Conc. concentration; gFW^{-1}, grams per fresh weight.

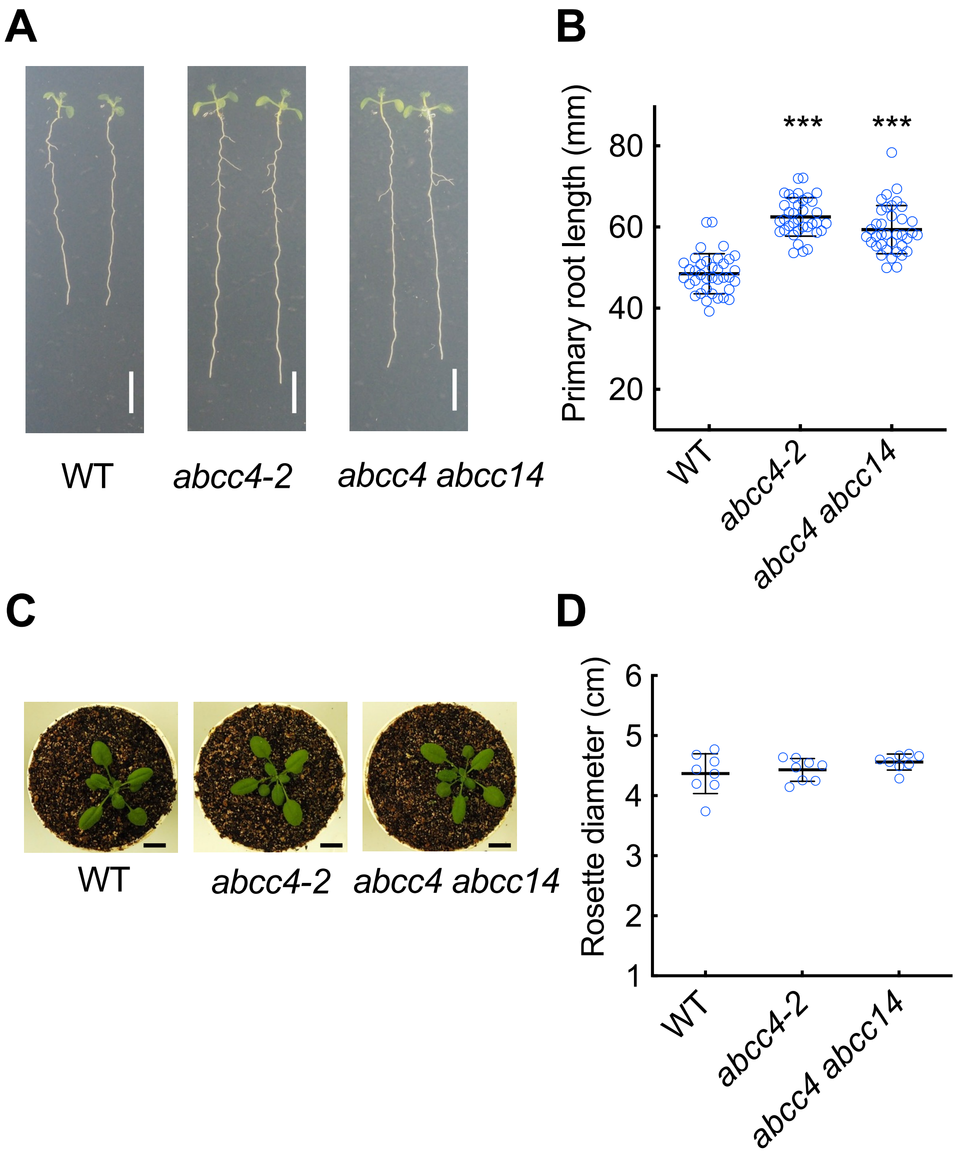

Supplementary Figure S9. Growth phenotype of *abcc4 abcc14*
(A) A representative image of WT (Col-0), *abcc4-2*, and *abcc4 abcc14* seedlings grown for 10 d. Scale bar, 1 cm. **(B)** Primary root length of WT and *abcc4 abcc14* seedlings grown for 10 d. Data are means ± SD (n_{WT}= 37, $n_{abcc4\text{-}2}$= 36, $n_{abcc4\ abcc14}$=38). Asterisks represent Student's *t*-test significance compared with WT (***$P <$ 0.001) **(C)** A representative image of WT, *abcc4-2*, and *abcc4 abcc14* grown for 23 d on soil. Scale bar, 1 cm. **(D)** Rosette diameter of WT, *abcc4-2*, and *abcc4 abcc14* grown for 23 d on soil. Data are means ± SD ($n = 8$). Asterisks in this figure represent Student's *t*-test significance compared with WT (***$P < 0.001$).

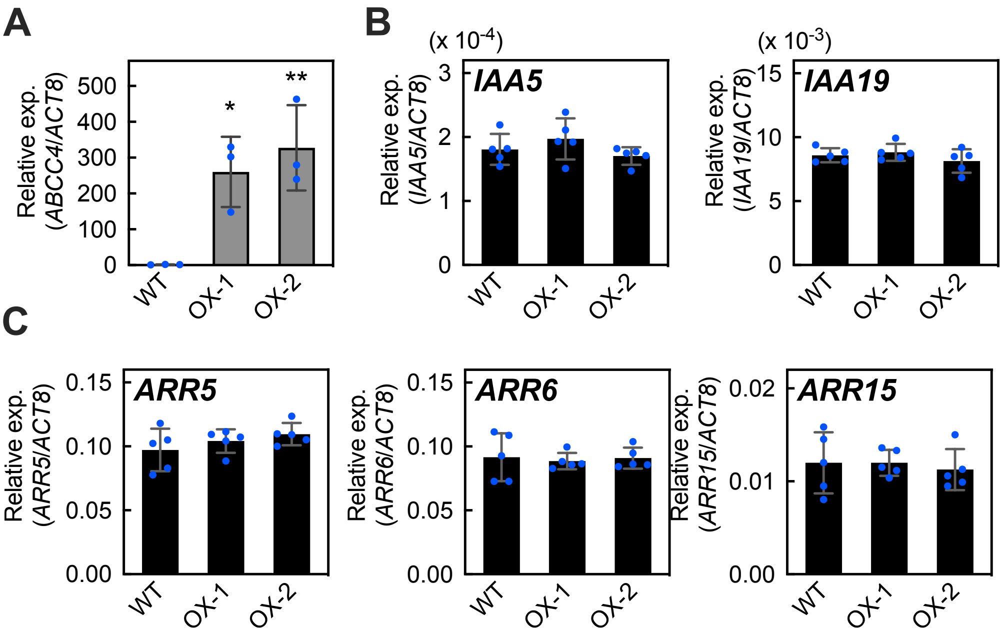

Supplementary Figure S10. Expression levels of cytokinin and auxin-responsive marker genes in *ABCC4* overexpressing lines

(A) Expression levels of *ABCC4* in two independent *ABCC4* overexpressing lines, OX-1 and OX-2. Whole seedlings grown for 10 d were harvested. Data are means ± SD (n = 3). **(B)** Expression levels of auxin-response marker genes (*IAA5* and *IAA19*) in WT (Col-0), OX-1, and OX-2 seedling roots grown for 10 d. Data are means ± SD (n = 5). **(C)** Expression levels of cytokinin-response marker genes (*ARR5, ARR6,* and *ARR15*) in WT, OX-1, and OX-2 seedling roots grown for 10 d. Data are means ± SD (n = 5). Expression levels were quantified by RT-qPCR analysis and normalized to *ACT8* as the internal control. Asterisks in this figure represent Student's *t*-test significance compared with WT (*$P < 0.05$, **$P < 0.01$).

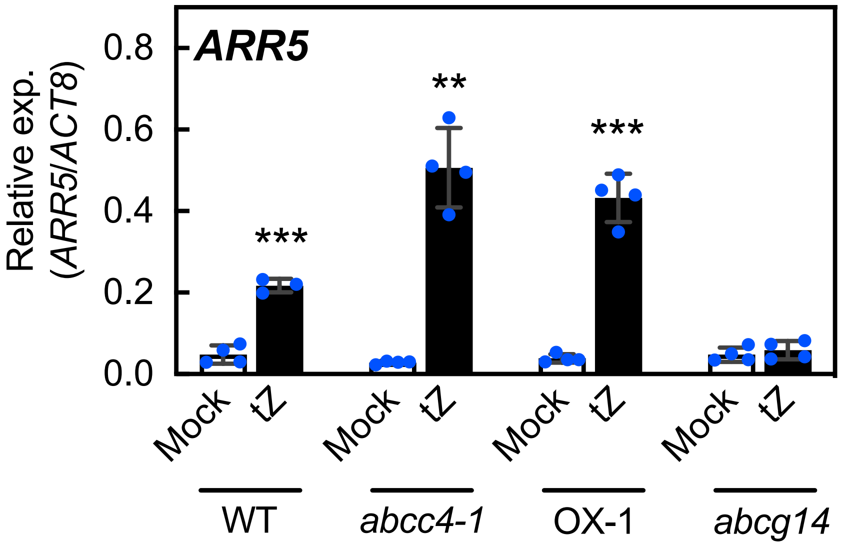

Supplementary Figure S11. *ARR5* expression levels in shoots followed by tZ application in roots in an *abcc4* mutant and an *ABCC4* overexpressing line

Expression levels of *ARR5* in shoots followed by 0.01 % DMSO (Mock) or 1 µM *trans*-zeatin (tZ) application to roots for 30 min were analyzed in WT (Col-0), *abcc4-1*, OX-1, and *abcg14* seedlings. Expression levels were quantified by RT-qPCR analysis and normalized to *ACT8* as the internal control. Data are means ± SD (n = 3~4). Asterisks represent Student's *t*-test significance compared with control of WT (**$P < 0.01$, ***$P < 0.001$).

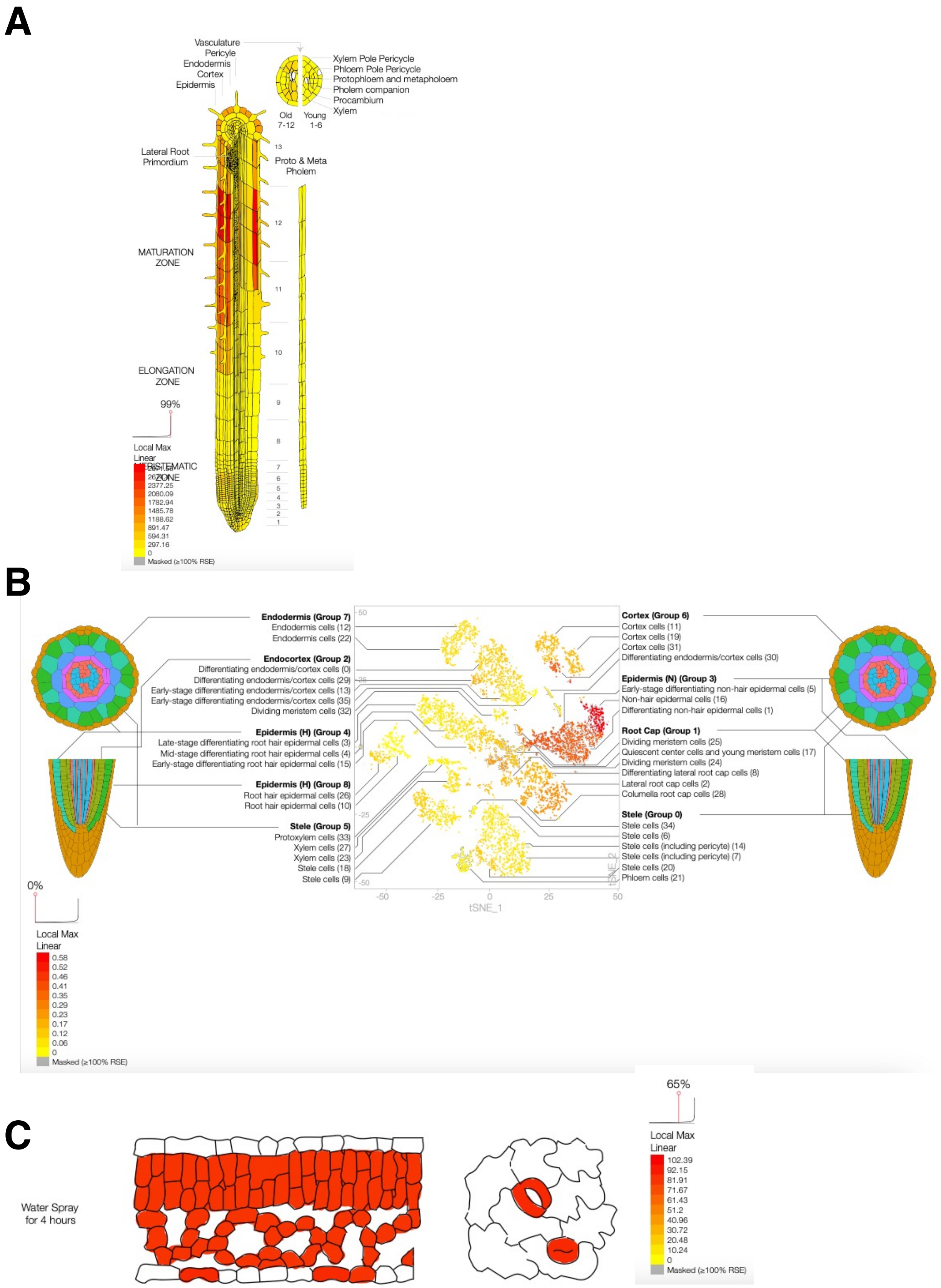

Supplementary Figure S12. Expression level of *ABCC4* according to the ePlant database (https://bar.utoronto.ca/eplant/).
ABCC4 expression pattern at tissue- **(A)** and single-cell levels **(B)** in root and mesophyll, and guard cells in rosette leaf **(C)**. Numbers on the right side of the heatmap indicate *ABCC4* expression intensity.

Supplementary Table S1. List of 61 candidate genes.

AGI	Gene Name
AT5G01240	LAX1 (LIKE AUXIN RESISTANT 1)
AT2G01420	PIN4 (PIN-FORMED 4)
AT1G77110	PIN6 (PIN-FORMED 6)
AT2G36910	ABCB1
AT3G28860	ABCB19
AT2G38120	AUX1
AT1G68100	IAR1 (IAA-ALANINE RESISTANT 1)
AT1G73590	PIN1 (PIN-FORMED 1)
AT1G25530	Transmembrane amino acid transporter family protein;(source:Araport11)
AT1G58360	AAP1 (AMINO ACID PERMEASE 1)
AT1G77380	AAP3 (AMINO ACID PERMEASE 3)
AT3G55740	PROT2 (PROLINE TRANSPORTER 2)
AT5G04770	CAT6 (CATIONIC AMINO ACID TRANSPORTER 6)
AT5G40780	LHT1 (LYSINE HISTIDINE TRANSPORTER 1)
AT5G49630	AAP6 (AMINO ACID PERMEASE 6)
AT4G27730	OPT6 (OLIGOPEPTIDE TRANSPORTER 1)
AT3G56200	Encodes a putative amino acid transporter
AT1G09960	SUC4 (SUCROSE TRANSPORTER 4)
AT1G19450	Major facilitator superfamily protein;(source:Araport11)
AT1G75220	ERDL6 (ERD6-LIKE 6)
AT4G16480	INT4 (INOSITOL TRANSPORTER 4)
AT5G18840	Major facilitator superfamily protein;(source:Araport11)
AT1G08930	ERD6 (EARLY RESPONSE TO DEHYDRATION 6)
AT1G11260	STP1 (SUGAR TRANSPORTER 1)
AT3G03090	VGT1 (VACUOLAR GLUCOSE TRANSPORTER 1)
AT3G18830	PMT5 (POLYOL/MONOSACCHARIDE TRANSPORTER 5)
AT4G35300	TMT2 (TONOPLAST MONOSACCHARIDE TRANSPORTER2)
AT1G21460	SWEET1
AT1G17840	ABCG11
AT1G51460	ABCG13
AT1G51500	ABCG12
AT1G67940	ABCI17
AT2G13610	ABCG5
AT2G26910	ABCG32
AT2G36380	ABCG34
AT2G37280	ABCG33
AT2G47800	ABCC4
AT3G21090	ABCG15
AT4G25750	ABCG4
AT5G52860	ABCG8
AT1G11670	MATE efflux family protein;(source:Araport11)
AT1G47530	DTX33
AT1G64820	MATE efflux family protein;(source:Araport11)
AT4G29140	ABS3 (ACTIVATED DISEASE SUSCEPTIBILITY 1)
AT1G61890	MATE efflux family protein;(source:Araport11)
AT3G23560	ALF5 (ABERRANT LATERAL ROOT FORMATION 5)
AT5G17700	MATE efflux family protein;(source:Araport11)
AT1G07290	GONST2 (GOLGI NUCLEOTIDE SUGAR TRANSPORTER 2)
AT1G10540	NAT8 (NUCLEOBASE-ASCORBATE TRANSPORTER 8)
AT1G52190	NRT1.11
AT3G16180	NRT1.12
AT3G47960	NPF2.10
AT1G54370	NHX5 (NA+/H+ ANTIPORTER 5)
AT1G79610	ATNHX6 (NA+/H+ ANTIPORTER 6)
AT3G05030	NHX2 (SODIUM HYDROGEN EXCHANGER 2)
AT5G13760	Plasma-membrane choline transporter family protein;(source:Araport11)
AT4G03560	CCH1 (CALCIUM CHANNEL 1)
AT1G80830	NRAMP1 (NATURAL RESISTANCE-ASSOCIATED MACROPHAGE PROTEIN 1)
AT4G24120	YSL1 (YELLOW STRIPE LIKE 1)
AT3G01280	VDAC1 (VOLTAGE DEPENDENT ANION CHANNEL 1)
AT2G38410	ENTH/VHS/GAT family protein;(source:Araport11)

Supplemenary Table S2. List of primers used for vector construction and genotyping.

Name (Forward/Reverse)	Purpose	Forward (5' to 3')	Reverse (5' to 3')
ABCC4-F/R	Construction of pENTR-ABCC4	CACCATGTGGTTGCTTTCGTCTTCTCC	TCATATTCCGGCAGATCGG
oxABCC4-F/R	Construction of pBA002-ABCC4	ACGGGGGACTCTAGAGGATCATGTGGTTGCTTTCGTCTTC	ATCGGGGAAATTCGAGCTCATCATATTCCGGCAGATCG
ABCC14--F/R	Construction of pENTR3C-ABCC4	CTTTAAAGGAACCAATTCAGATGCGGTGGCTTTCTTCTAC	GAAAGCTGGGTCTAGATATCTCATATTCCGGCAGATCG
gABCC4, 14-1-F/R	Construction of pMgPec12-137-2A-GFP-abcc4, 14	TTGGGTCTCGTGCAGAGGTAATAACCTTGCCACGGTTTTAGAGCTAGAAATAGCA	TTGGGTCTCCGATGGTAATACTTGCACCAGCCGGGAATCGAA
gABCC4, 14-2-F/R	Construction of pMgPec12-137-2A-GFP-abcc4, 14	TTGGGTCTCGCATCACAACCCAGTTTTAGAGCTAGAAATAGCA	TTGGGTCTCCTTACTACCTCGTTGCACCAGCCGGGAATCGAA
gABCC4, 14-3-F/R	Construction of pMgPec12-137-2A-GFP-abcc4, 14	TTGGGTCTCGGTAACCCTGTGAGTTTTAGAGCTAGAAATAGCA	TTGGGTCTCCAAACCGTTTTTGGCTGATGTTTCATGCACCAGCCGGGAATCGAA
rtABCC4—F/R	Genotyping of *abcc4* mutations	ATGTGGTTGCTTTCGTCTTC	TCATATTCCGGCAGATCGGA

Supplementary Table S3. List of primers used for RT-qPCR analysis.

Gene name	Locus ID	Forward (5' to 3')	Reverse (5' to 3')
ACT8	AT1G49240	AACATTGTGCTCAGTGGTGG	GTGGTGCCACGACCTTAATC
TIP41	AT4G34270	GTGAAAACTGTTGGAGAGAAGCAA	TCAACTGGATACCCTTTCGCA
ABCC4	AT2G47800	TTTAGCGCAGTGGATGCTCA	TGCAATCCACGTTGTGCAAG
ARR5	AT3G48100	GCTGTTGATGATAGTATGGTTG	AGATATTGTAAAGCTCTTGTCG
IAA5	AT1G15580	ATCTTGCTTCCGCTCTGCAA	ACGATCCAAGGAACATTTCCCA
IAA19	AT3G15540	TTTCCGTGGCATCGGTGTG	GCGAGCATCCAGTCTCCATC
