# Supplementary material for: The cytokinin efflux transporter ABCC4 participates in Arabidopsis root system development
Source: Plant Physiol. 2024 Dec 24;197(1):kiae628. doi: 10.1093/plphys/kiae628 (PMC11668331; doi:10.1093/plphys/kiae628)
Supplement: kiae628_Supplementary_Data [file pp.00759.2024-s05.pdf › pp.00759.2024-s05.pdf]

Plant Physiology®

Arabidopsis ABCC4 encodes a cytokinin efflux transporter and is involved in root system development

Takuya Uragami, Takatoshi Kiba, Mikiko Kojima, Yumiko Takebayashi, Yuzuru Tozawa, Yuki Hayashi, Toshinori Kinoshita, and Hitoshi Sakakibara

Review Timeline:

Submission Date:	14-May-2024
Editorial Decision:	24-Jun-2024
Revision Received:	24-Oct-2024
Accepted:	30-Oct-2024

24-Jun-2024

Prof. Hitoshi Sakakibara
Nagoya Daigaku
Nagoya 464-8601
Japan

RE: Arabidopsis ABCC4 encodes a cytokinin efflux transporter and is involved in root system development

Dear Dr. Sakakibara:

We have received reviews of your manuscript, which you may view at the end of this letter. On the basis of the advice received and my own evaluation, I believe that, pending revision, this article may ultimately be acceptable for publication in Plant Physiology.

The reviewers are enthusiastic about the new ABCC4 transporter identified but have also identified several areas where some additional experimentation is needed. First, there is agreement that more information is needed about tissue and subcellular expression of ABCC4 in relationship to the phenotypes dsecribed--I wonder if some of this could come from recent public datasets, including single cell analyses for gene expression. Second, of significance to the ABCC4 proposed function in cytokinin export, is some data confirming a change in cytokinin distribution in the mutant, potentially accomplished by measurement of cytokinin levels or by use of a TCS reporter to uncover changes in cytokinin activity.

When you are ready to submit your revision, click the below link and find the red arrow under your Author Tasks heading for this revision submission.

If you want to visit this version of the manuscript, you may click the link below and then click on the folder that reads "Post Decision Manuscripts".

Link Not Available

When uploading the revised version of this article, please be sure to include as complete an answer as possible to the referees' criticisms during the first round of reviews in the "response to reviewers" section.

Plant Physiology considers a revision for up to 60 days after a manuscript has been provisionally accepted; limited extension of this timeframe is at the discretion of the Monitoring Editor. A reminder notice will be sent to you 2 weeks before the deadline, at which time you may choose to contact the editorial office to request an extension; if so, please provide a justification for your request. After the expiration of this deadline, the article would need to be submitted as a resubmission and would be handled as such.

Thank you very much for giving us an opportunity to review this work. I look forward to receiving the next version.

Sincerely,

Editor, Plant Physiology

Want to add this revision deadline to your calendar? Click below!

-- Reviewer comments:
Reviewer #1 (Comments for the Author):

Although several classes of ck transporters have been described, our knowledge of cytokinin transportis incomplete. The authors used an innovative approach to screening over 60 putative transporters for ck export, and identified a novel ABCC4 transporter that effluxes active ck. The authors show that this protein can efflux active cks in both tobacco and yeast. This data

appears to be robust and support their claims.

They show that ABCC4 is expressed in roots (via RT-PCR) and that it is cytokinin inducible in this tissue. abcc4 knockouts have longer roots, and this can be rescued by adding cytokinin. Over expression leads to shorter roots and increased LR density. The phenotyping is well done, but the level of understanding on the expression/localization is very poor. Cytokinin levels are not significantly changed in these lines.

The authors hypothesize that alterations in ABCC4 lead to changes in cytokinin distribution. This needs to be checked. I appreciate that this is hard to do by measuring cytokinin directly, but one would predict that such a change would be observable by quantifying cytokinin readout in a cell specific manner using TCS.

The paper shows a clear phenotype for ABCC4 in regulating both stomatal opening and root meristem size. However, it is hard to understand what effect it may have in shaping an asymmetry in cytokinin distribution in these tissues. Central to this is understanding the cell type expression and sub cellular localization in these tissues. This feels like really important data that is missing. I appreciate that there is some data on this from the GUS line published in 2004, but I think this needs to be re-evaluated with a translational fusion in the specific tissues of interest. Although the expression and ck-inducibility in shoots was small, the guard cells make up a small proportion of the shoot material, so any specific changes in guard cells may be lost using bulk PCR approaches.

In conclusion, the authors identify a novel cytokinin transporter. They show two phenotypes associated with this. They relate the primary root growth to cytokinin; the stomata opening phenotype is less clearly linked with cytokinin. Given that they show ABCC4 effluxes cytokinin my opinion is that understanding changes in cytokinin distribution is essential to this manuscript. This cannot even be inferred due to lack of clarity on ABCC4 expression.

Reviewer #2 (Comments for the Author):

The manuscript by Uragami et al. is a significant contribution to our understanding of cytokinin transporters in root system development. Their biochemical data on the role of ABCC4 in mediating the efflux transport of cytokinins in tobacco cells and yeast cells, along with the physiological evidence from hormone profiling and phenotypic analysis, provide valuable insights. However, further investigation is needed to understand the tissue-specific expression pattern of ABCC4 in the roots and the mechanism of how ABCC4 affects cytokinin distribution.

The major concerns and comments are listed as follows:

1. Although the authors performed cytokinin transport activity assays in tobacco leaves and yeast, I suggest that they perform an efflux experiment in tobacco leaves or yeast cells in a time course.
2. The root meristem cells in Figure 5C are not quite clear. High-resolution images of the meristem cells are required.
3. The use of ProABCC4::GUS transgenic plants or immune staining methods to detect the expression level of ABCC4 in root tips could provide strong evidence to understand the role of ABCC4 in roots. If the authors have the genetic materials, I would suggest to show the distribution of GUS stain or green influence in the roots of abcc4 mutants or the overexpression lines by ARR5: GUS or TCSn: GFP reporter.

Minor revision

1. The reference of abcg14 mutant should be provided in the plant materials section.
2. Line 89: The description needs to be more accurate since no physiological data support that PUP1 and PUP2 exhibit cytokinin transport activity in planta.
3. L487 "ABCC4- overexpressing" and L542 "t-test", ABCC4 and t should be italic.
4. L542 the value of "n" for WT, abcc4-1 and abcc4-2 should be displayed seperately.

Editor Comments:

The reviewers are enthusiastic about the new ABCC4 transporter identified but have also identified several areas where some additional experimentation is needed. First, there is agreement that more information is needed about tissue and subcellular expression of ABCC4 in relationship to the phenotypes described--I wonder if some of this could come from recent public datasets, including single cell analyses for gene expression. Second, of significance to the ABCC4 proposed function in cytokinin export, is some data confirming a change in cytokinin distribution in the mutant, potentially accomplished by measurement of cytokinin levels or by use of a TCS reporter to uncover changes in cytokinin activity.

Response:

Thank you for your summarized comments and suggestions.

Regarding the point that more information is needed on the tissue and subcellular expression of *ABCC4* in relation to the described phenotypes, we conducted several experiments before our first submission. To determine the tissue- and cellular-level expression sites, we used *ABCC4 promoter:GUS* transcriptional fusion constructs and for subcellular localization, we employed *CaMV35S promoter:ABCC4:GFP* translational fusion constructs. However, these experiments did not yield substantial signals. More detailed explanations are provided in our response to the referees. A possible explanation for the lack of detectable signals with the *ABCC4 promoter:GUS* could be that the expression levels of the *ABCC4* gene were very low. Therefore, as suggested by the editor, we have added a discussion of the role of *ABCC4* using publicly available tissue-level and single-cell gene expression datasets. The extracted data from the ePlant database are shown in the newly added Supplementary Fig. S12 and a discussion on the possible role of *ABCC4* in root system development has been added (Lines 311 – 321, 328 – 342).

To further examine the effects of *ABCC4* loss-of-function and overexpression on cytokinin distribution/signaling, we generated transgenic *abcc4* and OX lines harboring *TCSn:GFP* and conducted microscopic observation of the fluorescence patterns in primary root. The results showed that the range of GFP fluorescence in the epidermal tissue of the root tips was significantly reduced in the overexpression lines compared to the WT. While the range in the *abcc4-1* was larger than that of WT, the difference was not statistically significant. This additional experiment provides evidence to support our finding that *ABCC4* modulates cytokinin distribution/signaling in the root tip region. We have added the results as Fig. 6, with the corresponding descriptions in the Results section (Lines 255 - 262). Based on our results and results from a previous study focusing on the role of cytokinin signaling in the root transition zone (Curr Biol, 2013, 23: 1812–1817; https://doi.org/10.1016/j.cub.2013.07.051), we have proposed

a possible role for *ABCC4* in primary root growth in the Discussion section (Lines 322 – 342).

As for the subcellular localization of ABCC4, the Klein et al. study (Plant J, 2004, 39: 219-236; https://doi.org/10.1111/j.1365-313X.2004.02125.x) determined the plasma membrane localization of ABCC4 (AtMRP4) using multiple experimental approaches. These included transient expression of ABCC4-GFP in onion epidermal cells and Arabidopsis cells, as well as immunodetection of ABCC4-GFP through subcellular fractionation using a sucrose density gradient. Based on the results generated using these robust and diverse methods, we would like to accept their conclusion regarding the plasma membrane localization of ABCC4.

Reviewer Comments:

Reviewer #1:

Although several classes of ck transporters have been described, our knowledge of cytokinin transport is incomplete. The authors used an innovative approach to screening over 60 putative transporters for ck export, and identified a novel ABCC4 transporter that effluxes active ck. The authors show that this protein can efflux active cks in both tobacco and yeast. This data appears to be robust and support their claims.

They show that ABCC4 is expressed in roots (via RT-PCR) and that it is cytokinin inducible in this tissue. abcc4 knockouts have longer roots, and this can be rescued by adding cytokinin. Over expression leads to shorter roots and increased LR density. The phenotyping is well done, but the level of understanding on the expression/localization is very poor. Cytokinin levels are not significantly changed in these lines.

Response:

This point is more specifically addressed in the following two comments, to which we have provided detailed responses.

The authors hypothesize that alterations in ABCC4 lead to changes in cytokinin distribution. This needs to be checked. I appreciate that this is hard to do by measuring cytokinin directly, but one would predict that such a change would be observable by quantifying cytokinin readout in a cell specific manner using TCS.

Response:

With respect to the role of *ABCC4* in cytokinin distribution/signaling, we generated transgenic *abcc4* and OX lines harboring *TCSn:GFP* and conducted microscopic observation of the

fluorescence patterns in primary root. The results showed that the range of GFP fluorescence in the epidermal tissue of the root tips was reduced in the overexpression lines compared to the WT. While the range in the *abcc4-1* was larger than that of WT, the difference was not statistically significant. This additional experiment provides evidence supporting our finding that *ABCC4* modulates cytokinin distribution/signaling in the root tip region. We have added the results as Supplementary Fig. 6, with the corresponding descriptions in the Results section (Lines 254 - 261). Based on the results from this and a previous study focusing on the role of cytokinin signaling in the root transition zone (Curr Biol, 2013, 23: 1812–1817; https://doi.org/10.1016/j.cub.2013.07.051), we have proposed a possible role for *ABCC4* in primary root growth in the Discussion section (Lines 322 – 342).

The paper shows a clear phenotype for ABCC4 in regulating both stomatal opening and root meristem size. However, it is hard to understand what effect it may have in shaping an asymmetry in cytokinin distribution in these tissues. Central to this is understanding the cell type expression and sub cellular localization in these tissues. This feels like really important data that is missing. I appreciate that there is some data on this from the GUS line published in 2004, but I think this needs to be re-evaluated with a translational fusion in the specific tissues of interest. Although the expression and ck-inducibility in shoots was small, the guard cells make up a small proportion of the shoot material, so any specific changes in guard cells may be lost using bulk PCR approaches.

Response:

After isolating the *ABCC4* gene, we aimed to determine its expression sites at the tissue and subcellular levels. First, we employed GUS as a reporter (*ABCC4pro-GUS*) and made two types of transcriptional fusion constructs for tissue and cellular level analysis: one using about 1,600 bp of the upstream region of the *ABCC4* (to avoid overlap with the adjacent structural gene) and another using about 2,000 bp of the upstream region of the *ABCC4* including part of the adjacent structural gene as described in the Klein et al. paper (Plant J, 2004, 39: 219-236; https://doi.org/10.1111/j.1365-313X.2004.02125.x). These constructs were introduced into *Arabidopsis*, and GUS staining was performed using the stable transformant lines. However, no staining was observed in either case. Regarding the subcellular localization of ABCC4, we used a translational fusion construct of GFP as a reporter (*CaMV35Spro-ABCC4:GFP*), making two types of constructs: one inserting GFP into the loop between 14th and 15th of the 16 transmembrane regions of ABCC4, as described in the Klein et al. paper. However, GFP fluorescence could not be detected by the tobacco leaf infiltration method or the mesophyll cell transient assay. In another approach, we inserted GFP into the loop between 7th and 8th, but no

fluorescence could be detected in the tobacco leaf infiltration experiment. Although we cannot definitively explain why we could not detect GFP expression in these experiments, a possible explanation for the lack of detectable signals with the *ABCC4 promoter:GUS* could be that the expression levels of the *ABCC4* gene were very low.

Thus, after exhausting the experimental options and failing to obtain substantial data, we followed the editor's advice to use publicly available tissue-level and single-cell analysis datasets. The extracted data from the ePlant database are shown in the newly added Supplementary Fig. S12 with the related descriptions added to the Discussion sections (Lines 311 – 321, 328 – 342).

As for the subcellular localization of ABCC4, the study by Klein et al. (Plant J, 2004, 39: 219-236; https://doi.org/10.1111/j.1365-313X.2004.02125.x) determined the plasma membrane localization of ABCC4 (AtMRP4) using multiple experimental approaches. These included transient expression of ABCC4-GFP in onion epidermal cells and Arabidopsis cells, as well as immunodetection of ABCC4-GFP through subcellular fractionation using a sucrose density gradient. Based on the results obtained using these robust and diverse methods, we would like to accept their conclusion regarding the plasma membrane localization of ABCC4.

As suggested by the reviewer, we isolated guard cells from rosette leaves and analyzed the expression of *ABCC4*. The results showed that while *ARR5* expression was clearly induced by cytokinin treatment, *ABCC4* expression was not inducible in guard cells, nor in whole leaves, suggesting that *ABCC4* expression in guard cells operates under a different regulatory mechanism compared to its regulation in roots. This result has been included as Supplemental Figure S3. (Lines 197 – 199)

In addition, we conducted an additional experiment to validate the effect of cytokinin on stomatal opening. The results showed that cytokinin treatment significantly increased stomatal aperture in the WT, while in the *abcc4-1* mutant, the size of stomatal aperture remained high under both mock and cytokinin treatments (Fig. 7B). No significant change was observed in the OX lines compared to the WT. This result suggests that cytokinin concentration in the guard cells of the *abcc4-1* mutant was elevated, causing stomata to remain maximally open, thus rendering them unresponsive to external cytokinin. In contrast, high cytokinin efflux activity in the OX lines likely counteracted the applied cytokinin, resulting in a lack of response. This result also suggests that cytokinin perception in guard cells is primarily intracellular, with receptors on the endoplasmic reticulum membrane mediating cytokinin action. We have added these results in Fig. 7 and incorporated related description into the Results (Lines 287 – 293) and Discussion (Lines 364 – 374) sections.

Reviewer #2:

The manuscript by Uragami et al. is a significant contribution to our understanding of cytokinin transporters in root system development. Their biochemical data on the role of ABCC4 in mediating the efflux transport of cytokinins in tobacco cells and yeast cells, along with the physiological evidence from hormone profiling and phenotypic analysis, provide valuable insights. However, further investigation is needed to understand the tissue-specific expression pattern of ABCC4 in the roots and the mechanism of how ABCC4 affects cytokinin distribution.

The major concerns and comments are listed as follows:

1. Although the authors performed cytokinin transport activity assays in tobacco leaves and yeast, I suggest that they perform an efflux experiment in tobacco leaves or yeast cells in a time course.

Response:

Following the reviewer's suggestion, we performed a time-course of cytokinin efflux analysis using the TSAL system. The results showed that the concentration of active forms (iP, tZ, and cZ) in the incubation buffer was significantly higher in the ABCC4-expressing line compared to the vector control from 1 to 6 h, with the difference between them being the highest from 6 to 12 h, and was still maintained at 24 h. The concentration of riboside and ribotide precursors was also elevated compared to the controls, but the difference was much lower than the active forms. The data for the active forms have been added as Fig. 1B, and the precursor results were added as Supplementary Fig. S1B.

We also observed cytokinin efflux in the empty vector control over time (Fig. 1B and Supplementary Fig. S1B). This phenomenon has been previously reported (Zhao et al., 2021, Front Plant Sci) and suggests that some experimental Agrobacterium strains possess the ability to produce cytokinins even in the absence of the biosynthesis gene on the Ti plasmid. This has been mentioned in the discussion section (Lines 298 – 302).

2. The root meristem cells in Figure 5C are not quite clear. High-resolution images of the meristem cells are required.

Response:

Following the reviewer's suggestion, we replaced Figure 5C with higher resolution images of root tips.

3. The use of ProABCC4::GUS transgenic plants or immune staining methods to detect the expression level of ABCC4 in root tips could provide strong evidence to understand the role of ABCC4 in roots. If the authors have the genetic materials, I would suggest to show the distribution of GUS stain or green influence in the roots of abcc4 mutants or the overexpression lines by ARR5: GUS or TCSn: GFP reporter.

Response:

After isolating the *ABCC4* gene, we aimed to determine its expression sites at the tissue and subcellular levels. First, we employed GUS as a reporter (*ABCC4pro-GUS*) and made two types of transcriptional fusion constructs for tissue and cellular level analysis: one using about 1,600 bp of the upstream region of the *ABCC4* (to avoid overlap with the adjacent structural gene) and another using about 2,000 bp of the upstream region of the *ABCC4* including part of the adjacent structural gene as described in the Klein et al. paper (Plant J, 2004, 39: 219-236; https://doi.org/10.1111/j.1365-313X.2004.02125.x). These constructs were introduced into *Arabidopsis*, and GUS staining was performed using the stable transformant lines. However, no staining was observed in either case. Regarding the subcellular localization of ABCC4, we used a translational fusion construct of GFP as a reporter (*CaMV35Spro-ABCC4:GFP*), making two types of constructs: one inserting GFP into the loop between 14th and 15th of the 16 transmembrane regions of ABCC4, as described in the Klein et al. paper. However, GFP fluorescence could not be detected by the tobacco leaf infiltration method or the mesophyll cell transient assay. In another approach, we inserted GFP into the loop between 7th and 8th, but no fluorescence could be detected in the tobacco leaf infiltration experiment. Although we cannot definitively explain why we could not detect GFP expression in these experiments, a possible explanation for the lack of detectable signals with the *ABCC4 promoter:GUS* could be that the expression levels of the *ABCC4* gene were very low.

Thus, after exhausting the experimental options and failing to obtain substantial data, we followed the editor's advice to use publicly available tissue-level and single-cell analysis datasets. The extracted data from the ePlant database are shown in the newly added Supplementary Fig. S12 with the related descriptions added to the Discussion sections (Lines 311 – 321, 328 – 342).

With respect to the role of *ABCC4* in cytokinin distribution/signaling, we generated transgenic *abcc4* and OX lines harboring *TCSn:GFP* and conducted microscopic observation of the

fluorescence patterns in primary root. The results showed that the range of GFP fluorescence in the epidermal tissue of the root tips was reduced in the overexpression lines compared to the WT. While the range in the *abcc4-1* was larger than that of WT, the difference was not statistically significant. This additional experiment provides evidence supporting our finding that *ABCC4* modulates cytokinin distribution/signaling in the root tip region. We have added the results as Fig. 6, with the corresponding descriptions in the Results section (Lines 255 - 262). Based on the results from this and a previous study focusing on the role of cytokinin signaling in the root transition zone (Curr Biol, 2013, 23: 1812–1817; https://doi.org/10.1016/j.cub.2013.07.051), we have proposed a possible role for *ABCC4* in primary root growth in the Discussion section (Lines 322 – 342).

Minor revision

1. The reference of abcg14 mutant should be provided in the plant materials section.

Response:

We have added the description of *abcg14* mutant with reference in the plant materials section (Line 386).

2. Line 89: The description needs to be more accurate since no physiological data support that PUP1 and PUP2 exhibit cytokinin transport activity in planta.

Response:

As you pointed out, PUP1 and PUP2 were reported to mediate cytokinin uptake in a heterologous yeast system, but their physiological role is not clear. Therefore, we have described PUP1 and PUP2 in a separate sentence to be more precise (Lines 99 – 101).

3. L487 "ABCC4- overexpressing" and L542 "t-test", ABCC4 and t should be italic.

Response:

We have italicized the words.

4. L542 the value of "*n*" for WT, *abcc4-1* and *abcc4-2* should be displayed separately.

Response:

According to your suggestion, we have displayed each value of "*n*" separately in the legend.

30-Oct-2024
Prof. Hitoshi Sakakibara
Nagoya Daigaku
Nagoya 464-8601
Japan

MSID: PP2024-RA-00759R1
MS TITLE: Arabidopsis ABCC4 encodes a cytokinin efflux transporter and is involved in root system development

Dear Dr. Hitoshi Sakakibara:

I am pleased to inform you that your revised manuscript "Arabidopsis ABCC4 encodes a cytokinin efflux transporter and is involved in root system development" has been accepted for publication in Plant Physiology. Your manuscript will be checked for consistency with journal formatting and image standards by one of our Science Editors, who will contact you shortly. They may have a number of queries and suggestions for you. Please keep in mind that they work to increase the online visibility of your article and its attraction for readers from the widest of backgrounds. You will have the opportunity to review any revisions, and I hope you will work constructively with them to get the most out of your research. You will also have the opportunity to review all changes when you receive the author proofs for your article.

Once validated for publication, your article will be sent to a Science Editor for a final review of your manuscript and source files. After your final files are uploaded, your article will appear in the next available issue and the online Preview version of your article will be posted after your signed Copyright and Checklist forms and final source files are received.

Please be sure to include the following in your manuscript file:
Harvard style citations
List of all author contributions and funding information at the beginning of manuscript after the titles and author list.
List all supplemental materials associated with the article, with titles, at the end of the ms, before the references and acknowledgments section.
Complete Figure Legends for all figures appearing in the article listed before the Literature Cited.

LICENSE INFORMATION Shortly after your paper is sent to production, you will receive an email from our publisher's author support team, **SciPris**, with information on article processing charges, as well as the types of Open Access license you would be able to purchase. The accepted version of your manuscript will not appear on our Advance Articles page until the license is selected and signed, so please look out for an email from SciPris. For more information see the Article Publication Fees section of our author guidelines: https://academic.oup.com/plcell/pages/General-Instructions

Finally, we encourage your submission of artwork for the journal cover. If there is an image or illustration related to your paper that you would like to have considered, please email it in .pdf or .tif format to clowe@aspb.org. An image title, brief, 2-3 sentence description of the image, and the name of the person credited for the image are also encouraged. If your image is selected to appear on the online cover of the journal, you will be contacted with the scheduled issue date and further information.

Thank you and congratulations on your Plant Physiology paper!

Sincerely,
Editor, Plant Physiology

=====
FIRST AUTHOR PROFILES
=====

We recognize the hard work that goes into being First Author of a Plant Physiology paper by publishing a "First Author Feature" for each First Author alongside your paper.

If the first author(s) would like to submit information for a feature on Facebook and Twitter, fill out the form by clicking on the link below:

Plant Physiology First Author Feature Form: https://forms.office.com/r/ZBCGZfhm8F
If you cannot access the form through the link above, please contact Rachel at rbelsky@aspb.org.

To upload an image of yourself to accompany your profile, please put your photo in the correct journal folder at the link below.
You must label your image: Author First Name Last Name Article Title.

First Author Profile Image Dropbox: https://www.dropbox.com/scl/fo/xz029p7qjr2jml2rp2kmq/h?dl=0&rlkey=6fkxcaqmxx35ap152b2osy1fz

=====
IMPORTANT REMINDER: PEER REVIEW REPORTS
=====

If you opted to publish a peer review report along with your article during the original submission process, it will be prepared by the editorial staff and publicly posted with your manuscript, inside the zip file that contains any other supplemental material. As a reminder, the peer review report is a public record of all comments from editors and reviewers, as well as your prior responses, as you received them in the decision letters for each draft of your manuscript. If you agreed to publish this report and have changed your mind, or are not sure if you selected this option, please contact the editorial office as soon as possible before signing the license agreement from our publisher.

:
